# Sequential Immunization With Live-Attenuated Chimeric Hemagglutinin-Based Vaccines Confers Heterosubtypic Immunity Against Influenza A Viruses in a Preclinical Ferret Model

**DOI:** 10.3389/fimmu.2019.00756

**Published:** 2019-04-10

**Authors:** Wen-Chun Liu, Raffael Nachbagauer, Daniel Stadlbauer, Alicia Solórzano, Francesco Berlanda-Scorza, Adolfo García-Sastre, Peter Palese, Florian Krammer, Randy A. Albrecht

**Affiliations:** ^1^Department of Microbiology, Icahn School of Medicine at Mount Sinai, New York, NY, United States; ^2^Global Health and Emerging Pathogens Institute, Icahn School of Medicine at Mount Sinai, New York, NY, United States; ^3^PATH US, Seattle, WA, United States; ^4^Department of Medicine, Icahn School of Medicine at Mount Sinai, New York, NY, United States

**Keywords:** universal influenza virus vaccine, live-attenuated influenza vaccine, ferret, stalk antibody, chimeric hemagglutinin, heterosubtypic protection

## Abstract

Due to continuous antigenic drift and occasional antigenic shift, influenza viruses escape from human adaptive immunity resulting in significant morbidity and mortality in humans. Therefore, to avoid the need for annual reformulation and readministration of seasonal influenza virus vaccines, we are developing a novel chimeric hemagglutinin (cHA)-based universal influenza virus vaccine, which is comprised of sequential immunization with antigens containing a conserved stalk domain derived from a circulating pandemic H1N1 strain in combination with “exotic” head domains. Here, we show that this prime-boost sequential immunization strategy redirects antibody responses toward the conserved stalk region. We compared the vaccine efficacy elicited by distinct vaccination approaches in the preclinical ferret model of influenza. All ferrets immunized with cHA-based vaccines developed stalk-specific and broadly cross-reactive antibody responses. Two consecutive vaccinations with live-attenuated influenza viruses (LAIV-LAIV) conferred superior protection against pH1N1 and H6N1 challenge infection. Sequential immunization with LAIV followed by inactivated influenza vaccine (LAIV-IIV regimen) also induced robust antibody responses. Importantly, the LAIV-LAIV immunization regimen also induced HA stalk-specific CD4^+^IFN-γ^+^ and CD8^+^IFN-γ^+^ effector T cell responses in peripheral blood that were recalled by pH1N1 viral challenge. The findings from this preclinical study suggest that an LAIV-LAIV vaccination regimen would be more efficient in providing broadly protective immunity against influenza virus infection as compared to other approaches tested here.

## Introduction

Type A influenza viruses (both H1N1 and H3N2 subtypes) and type B influenza viruses continue to co-circulate globally in humans causing seasonal epidemics that result in morbidity and mortality worldwide (1). Current licensed seasonal influenza virus vaccines formulated as inactivated influenza virus vaccine (IIV), live-attenuated influenza virus vaccines (LAIV), or recombinant hemagglutinin (HA) proteins are proven to protect against infection by influenza virus (2), but they only provide suboptimal protection against infection by seasonal influenza viruses (ranging from 10 to 60%) (3). Antigenic shift or drift of the surface glycoproteins [HA and neuraminidase (NA)] can result in an antigenic mismatch between the vaccine strain and circulating strains which leads to substantial decreases in vaccine effectiveness (4). To match the continuous antigenic evolution of influenza viruses, current seasonal influenza virus vaccines are reformulated and readministered annually (5) based on the influenza virus vaccine strains selected by the World Health Organization. Although current influenza virus vaccines induce protection against infection by antigenically similar influenza viruses, they provide suboptimal protection against unpredictable emerging pandemic strains. Once a pandemic virus emerges, it takes ~5–6 months from vaccine strain selection to distribution of a strain-matched pandemic vaccine (6). During this time frame, the population may be left vulnerable.

To mitigate these concerns, there is a strong public health interest to develop a broadly protective or universal influenza virus vaccine to induce durable and broadly cross-reactive antibody responses, which would confer cross-protection against all influenza viruses and would alleviate potential disease burden once a pandemic influenza virus strain emerges (3, 7, 8). Designing immunogens and vaccination strategies that focus the humoral immune response on the highly conserved HA stalk region is regarded as a promising strategy to achieve this goal. Compared to current, licensed seasonal influenza virus vaccines which predominately induce potent strain-specific antibodies against the immunodominant HA head domain (9–11), several innovative vaccine candidates were proven to induce protective antibody responses against the immuno-subdominant HA stalk domain (12, 13) and confer heterosubtypic protection in mice (14–17) and ferrets (17–19). Toward the goal of a universal influenza virus vaccine, we have developed group 1 hemagglutinin-specific influenza virus vaccines based on chimeric HAs (cHAs) composed of a stalk domain derived from a circulating influenza virus strain and head domains from “exotic” subtypes of influenza viruses that do not circulate in humans. Through sequential immunizations, repeated exposure to these cHAs redirects antibody responses toward the conserved stalk domain (20–22). Importantly, most adults have pre-existing stalk antibodies from prior influenza virus infection or vaccination so it is expected that our immunization approach will boost anti-stalk antibody responses after one or two vaccinations with cHAs (10, 23, 24). Recent reports also indicated that LAIV could successfully prime immune responses against infection by exotic viruses (25–27). Our recent study demonstrated that a cHA-based vaccination regimen with a prime immunization with LAIV followed by booster immunization with Adjuvant System 03 (AS03)-adjuvanted inactivated split influenza virus (LAIV-IIV) vaccine conferred superior protection against 2009 pandemic H1N1 (pH1N1) infection compared to two doses of AS03-adjuvanted split influenza virus (IIV-IIV) vaccine in the preclinical ferret model (18). Due to comparable antibody responses between LAIV-IIV and IIV-IIV regimens, we hypothesized that cell-mediated immunity may also contribute to protection. The vaccination regimens that we selected from our previous study, i.e., cH8/1N1 LAIV followed by cH5/1N1 IIV w/or w/o AS03 adjuvant and cH8/1N1 IIV followed by cH5/1N1 IIV w/ AS03 adjuvant, have advanced to a human Phase I trial (*ClinicalTrials.gov* # NCT03300050).

Nasal spray delivery of LAIV vaccines was reported to elicit stronger and longer-lasting immunity (both robust B-cell and T-cell responses) than IIV vaccination in children (28, 29). In this preclinical study, we compared one or two dose regimen of cHA-based LAIV vaccines to a LAIV-IIV vaccination regimen to provide protective immunity against pH1N1 virus challenge. Moreover, we also examined the breadth of protective immunity induced by our sequential immunization approach against challenge by a heterosubtypic H6N1 virus encoding an antigenically distinct HA stalk. Our results demonstrated that our sequential immunization approach with a cHA-based LAIV-LAIV vaccination regimen afforded the best protection against pH1N1 and H6N1 influenza virus infections, induced broadly cross-reactive and HA stalk-specific antibody responses, and could induce HA stalk-specific T cell immunity.

## Results

### Chimeric HA-Based LAIV-LAIV and LAIV-IIV Vaccination Regimens Induce Stalk-Reactive Immunity in Ferrets

**Figure 1:**
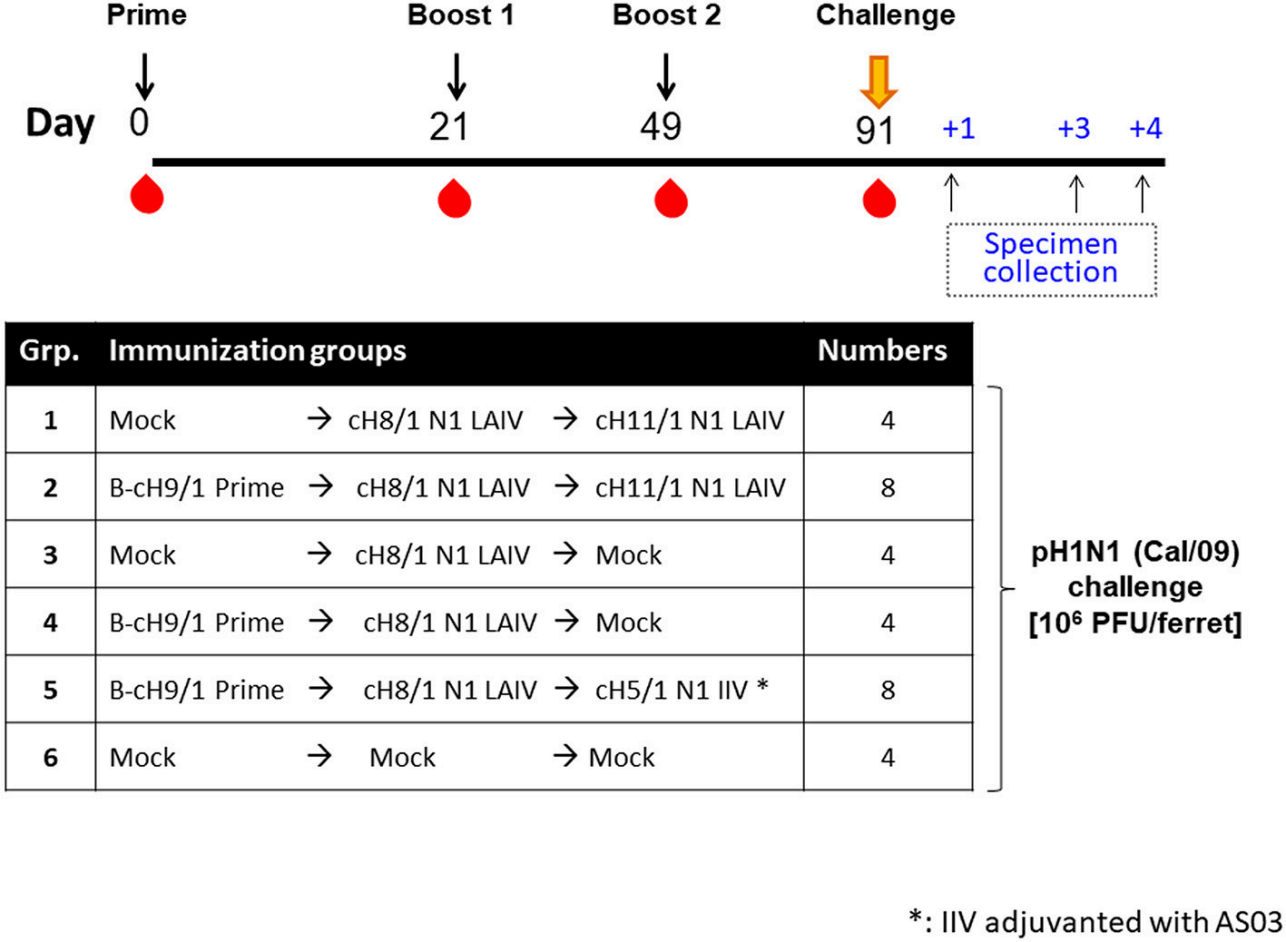
Overview of the pH1N1 viral challenge study design. The timeline and experimental groups for the pH1N1 challenge study are outlined. Ferrets in the cHA vaccination groups were unprimed or primed with an influenza B-cH9/1 virus. All cHA vaccination groups except for the mock animals received a booster immunization of cH8/1 LAIV on day 21, followed by a second booster immunization on day 49 with cH11/1 LAIV or cH5/1 IIV vaccine (with adjuvant AS03) to boost stalk-specific antibodies. Mock immunization controls received plain allantoic fluid (mock group). All ferrets were challenged with pH1N1 (Cal/09) influenza A virus on day 91, and sample specimens were collected on days 1, 3, and 4 post-challenge infection.

The objective of this preclinical study was to assess the protective immunity induced by immunization with group 1 hemagglutinin-specific influenza virus vaccines based on chimeric hemagglutinins (cHAs) in a ferret model of influenza. The experimental designs and immunization strategies are summarized in Figure 1. Since most human adults have a primed repertoire of B cells with specificity to the HA stalk domain (19, 30), we included an influenza B virus expressing cH9/1 (B-cH9/1) to mimic pre-existing HA stalk immunity. We then compared the ability of our sequential immunization strategies to boost antibody titers against the group 1 hemagglutinin stalk (Figure 1). LAIV vaccines were reported to induce more robust humoral and cellular immune responses in children (28, 29), but not in adults (31), and the results of our previous study (19) demonstrated that LAIV followed by AS03 adjuvanted-split inactivated influenza virus vaccines (IIV) [LAIV-IIV] and IIV-IIV vaccination regimens effectively induced stalk-specific immunity in ferrets. To see whether LAIV alone may be sufficient to induce protective immunity, we included immunization groups that received one or two doses of cHA-based LAIV vaccines. The LAIV strains used in this study were first characterized by a pathotyping pilot study. Our results showed that the cH11/1 N1 LAIV (cH11/1 LAIV) virus is attenuated in ferrets as compared to wild-type A/California/04/09 (Cal/09) virus in terms of absence of body weight loss and undetectable viral replication in the upper and lower respiratory tract as shown in our previous study (cH8/1 LAIV) (19) and in Figure S1 (cH11/1 LAIV). cH8/1 LAIV and cH11/1 LAIV vaccine strains were selected for this study as the first and second booster immunizations, respectively. We excluded the cH5/1 LAIV component in the vaccination regimen to avoid potential safety concerns over administering an H5 virus to humans. In addition, since our previous studies demonstrated that addition of the AS03 adjuvant to the cHA-based influenza split IIV vaccine substantially improved antibody titers against the HA stalk (both IgG and IgA in serum) and against the N1 neuraminidase (NA) in mouse (32) and ferret models (19), AS03 was included in all IIV vaccines in this preclinical study.

**Figure 2:**
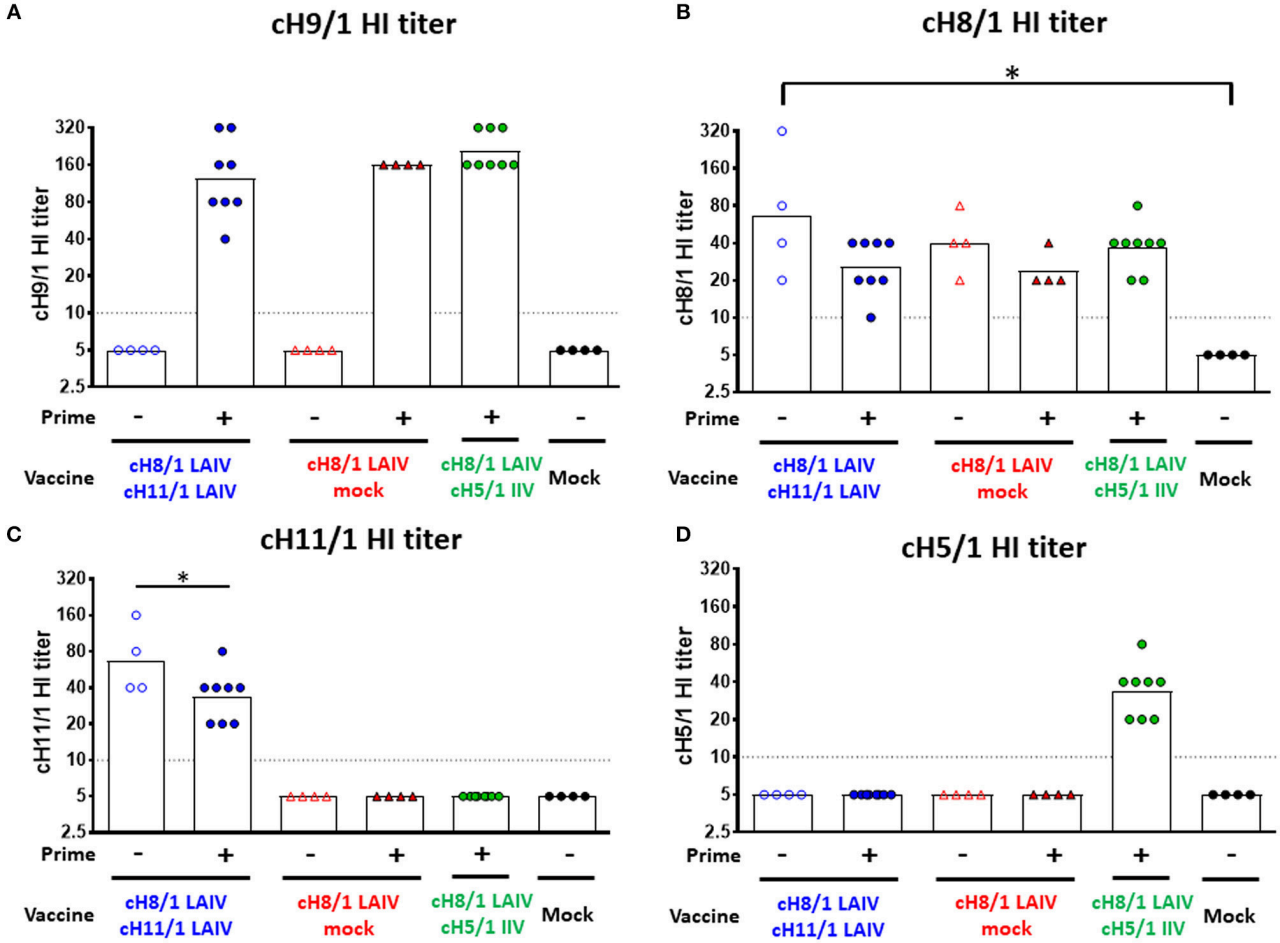
Vaccine-specific antibody titers measured by hemagglutination inhibition assay. Pre-challenge (day 91) hemagglutination inhibition (HI) titers against **(A)** cH9/1, **(B)** cH8/1, **(C)** cH11/1, and **(D)** cH5/1 viruses were measured prior to pH1N1 influenza A virus challenge infection. Y-axis indicates HI titers against the different virus strains. LAIV-LAIV vaccinated animals are shown as blue circles. Single dose of LAIV vaccinated animals are shown as red triangles. LAIV followed by AS03-adjuvanted IIV vaccinated animals are shown as green circles. Mock-immunized animals are shown as black circles. Empty symbols denote unprimed. Closed symbols denote B-cH9/1 virus prime immunization. White bars indicate the geometric mean titers (GMT) with individual scatter dot plots. Each point indicates the titer for each individual ferret (*n* = 4 or 8 /group). The black dashed line indicates the limit of detection for the assay. Data were analyzed by one-way ANOVA with Tukey multiple comparisons test. The asterisks refer to the level of significance. ^*^p < 0.05.

Four-month-old neutered male ferrets were initially primed with a replication competent influenza B virus expressing cH9/1 (B-cH9/1 live virus), followed by booster vaccination(s) with cH8/1 LAIV, cH8/1 LAIV followed by cH11/1 LAIV (LAIV-LAIV), or cH8/1 LAIV followed by AS03-adjuvanted cH5/1 IIV (LAIV-IIV). Since human adults have a primed HA stalk-specific B-cell repertoire (23, 30), ferrets that were not primed with the B-cH9/1 live virus were also included in the experimental design to examine the impact of pre-existing H1 stalk immunity. To assess the protective efficacy of these vaccination regimens, the ferrets were challenged with pH1N1 (Cal/09) virus. Sera were collected at the indicated time points and vaccine-induced antibody responses were evaluated (Figure 1). Hemagglutination inhibition (HI) titers were measured on day 91 (pre-challenge). Ferrets immunized with the influenza B-cH9/1 virus developed H9-specific HI titers ranging from 40 to 320 (Figure 2A). All animals which received the booster cH8/1 LAIV vaccination developed H8-specific HI titers ranging from 10 to 320 (Figure 2B). Following the second booster immunization with cH11/1 LAIV or cH5/1 IIV, the ferrets developed H11-specific or H5-specific HI titers that ranged from 20 to 160 or 20 to 80, respectively (Figures 2C,D). Importantly, during the course of the immunization schedule, all ferrets remained serologically negative for the pH1N1 influenza virus (Figure S2A).

**Figure 3:**
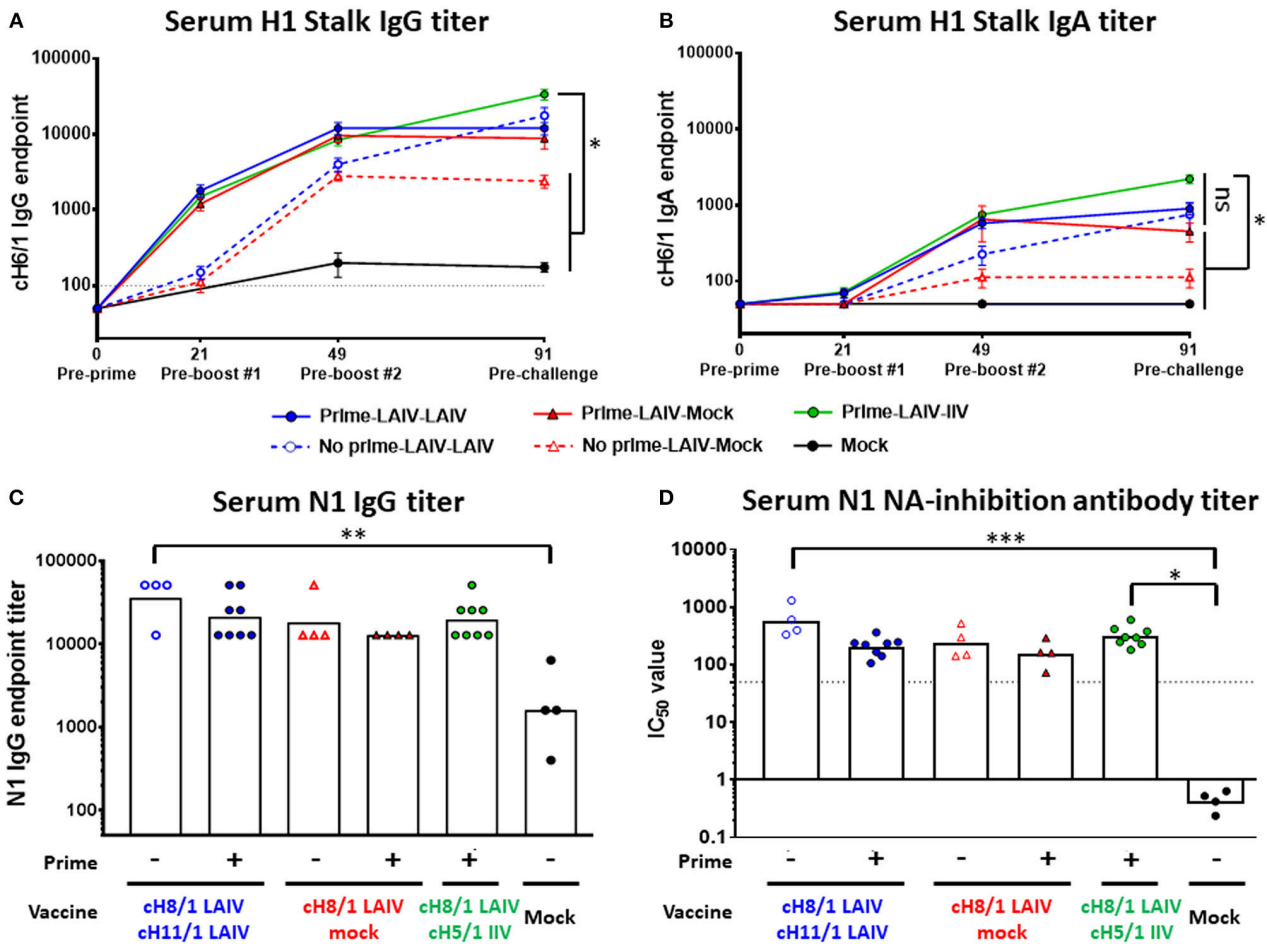
H1 stalk- and N1-specific antibody titers measured by ELISA and/or ELLA. The IgG antibody responses were measured on days 0, 21, 49, and/or 91. **(A)** H1 stalk serum IgG and **(B)** H1 stalk serum IgA mean endpoint titers ± standard error of the mean (SEM) against cH6/1 protein were indicated. **(C)** N1 serum total IgG titer against N1 (Cal/09) protein by ELISA is plotted on the y-axis. Each point indicates the GMT for each individual animal (*n* = 4 or 8/ group). The white bars indicate the averaged endpoint titer of each experimental group. The black dashed line indicates the limit of detection for the assay. **(D)** Fetuin-based enzyme-linked lectin assays were used to measure NA-inhibiting-antibody titers as reductions in the N1 NA enzymatic activities of the H7N1 viruses. The percentage of NA-inhibiting curves were graphed in Figure S2C. Corresponding IC_50_ values were determined as the 50% reduction in the NA enzymatic activities of H7_mal_N1_Cal09_ virus strain. Data in **(A,B)** were analyzed by two-way ANOVA followed by a Tukey's multiple comparison test (multiple time points). Data in **(C,D)** were compared to mock vaccinated animals with one-way ANOVA followed by a Dunnett's multiple comparison test (single time point). ns, no significant difference. The asterisks refer to the level of significance: ^*^p < 0.05; ^**^p < 0.01; ^***^p < 0.001.

The kinetics of H1 HA stalk-specific antibody responses were measured by enzyme-linked immunosorbent assay (ELISA). B-cH9/1 virus-primed ferrets induced anti-H1 stalk IgG titers of 1:1100 to 1:1600 at day 21 (pre-boost #1). At day 49 (pre-boost #2), anti-stalk antibodies were boosted in animals that received one dose of LAIV vaccines. The B-cH9/1 virus primed groups generally had 3-fold higher stalk-specific antibody titers than the unprimed groups (Figure 3A). Notably, for the Prime-LAIV-Mock and Prime-LAIV-LAIV groups, immunization with cH8/1 LAIV boosted stalk-specific IgG titers, which reached a plateau (~1:10,000) at day 49 and remained unchanged following the second booster immunization (day 91). In the absence of the B-cH9/1 prime immunization, sequential immunization with LAIV-LAIV induced anti-stalk IgG titers that were similar to those induced by sequential immunization with Prime-LAIV-LAIV on day 91, with a geometric mean titer (GMT) of 1:15,222. Sequential immunization with Prime-LAIV-IIV vaccines induced the highest H1 stalk-specific IgG titers which peaked on day 91 at a titer of 1:30,444 (Figure 3A). In addition to the H1 stalk-reactive IgG responses, we also monitored serum anti-stalk IgA titers induced by these different vaccination regimens. The serum stalk-reactive IgA responses shared a similar trend as the serum IgG response albeit at one log lower titers. Although the Prime-LAIV-IIV immunization induced the highest serum anti-H1 stalk IgA responses, there were no significant differences in the H1 stalk-reactive IgA antibody titers induced by the Prime-LAIV-LAIV, Prime-LAIV-IIV, and Mock-LAIV-LAIV vaccination regimens (Figure 3B). Nasal IgA was reported to correlate with protection against influenza virus infection in human cohorts (33), especially for nasal delivered vaccines such as LAIV; therefore, we also measured the anamnestic stalk-specific mucosal IgA antibody titers in nasal washes on day 3 post-pH1N1 (Cal/09) influenza virus challenge infection. All vaccinated ferrets, except for the mock-immunized animals, developed nasal IgA antibody responses, albeit, varied titers were observed within each immunization group (Figure S2B). The LAIV-LAIV and Prime-LAIV-LAIV vaccination regimens induced greater anti-N1 NA IgG antibody levels as compared to the mock immunization (Figure 3C). Similar to the pattern of anti-N1 IgG titers, elevated anti-N1 neuraminidase inhibition titers were also observed in all cHA vaccinated groups (Figure 3D, Figure S2C). Notably, LAIV-LAIV and Prime-LAIV-IIV induced significantly higher anti-N1NA inhibiting antibody titers than the other vaccinated groups, with GMT of IC_50_ value of 566 and 310, respectively (Figure 3D).

**Figure 4:**
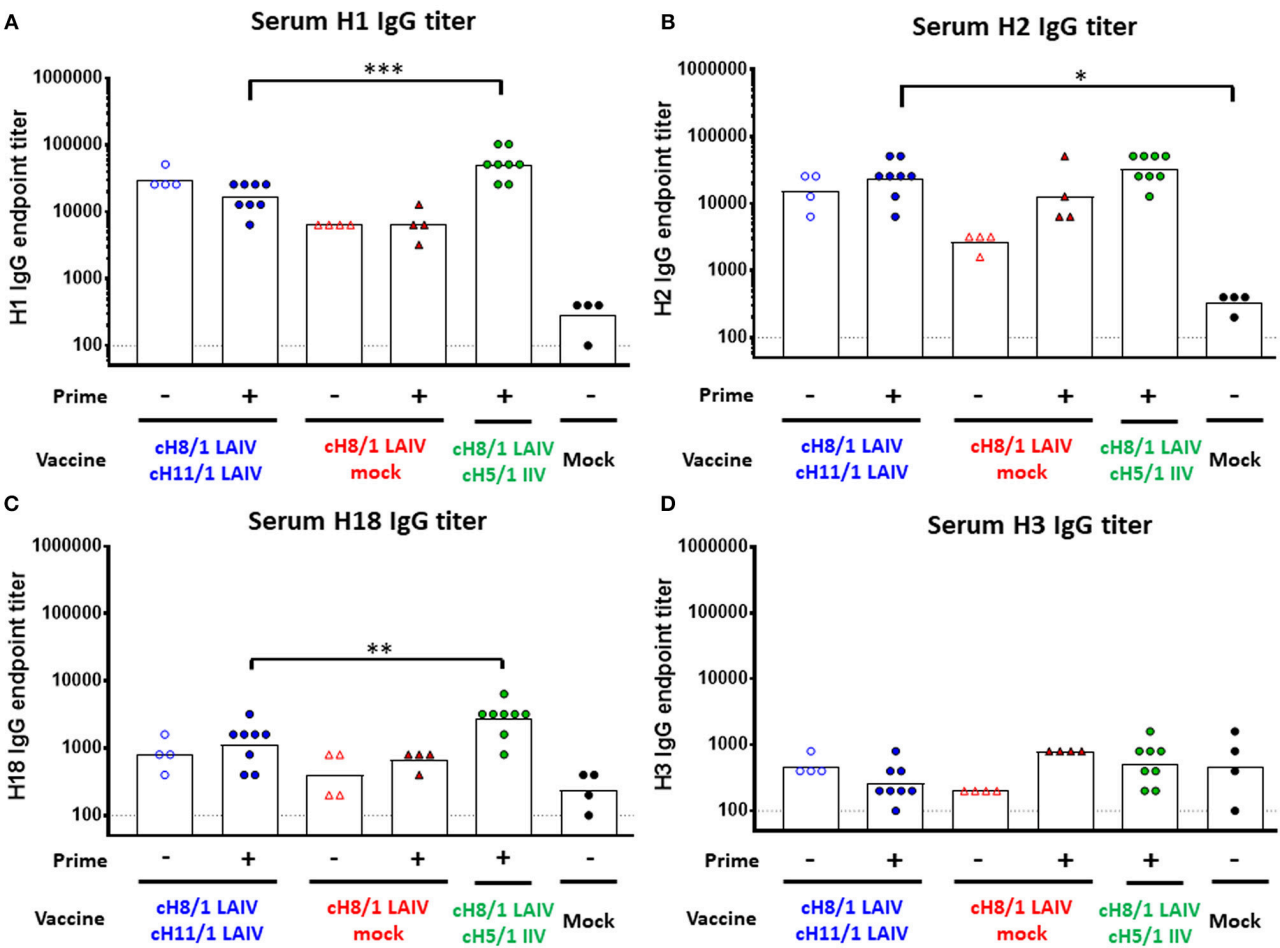
Breadth of antibody responses measured by ELISA. Serum IgG endpoint titers (y-axis) against group 1 **(A)** H1, **(B)** H2, **(C)** H18, and group 2 **(D)** H3 were measured prior to pH1N1 challenge infection (day 91). LAIV-LAIV vaccinated animals are shown as blue circles. Single dose of LAIV vaccinated animals are shown as red triangles. LAIV followed by AS03-adjuvanted IIV vaccinated animals are shown as green circles. Mock-immunized animals are shown as black circles. Empty symbols denote unprimed. Closed symbols denote B-cH9/1 prime immunization. White bars indicate the GMT with individual scatter dot plots. Each point indicates the endpoint titer for each individual ferret (*n* = 4 or 8/group). Data were compared to Prime-LAIV-LAIV vaccinated animals with one-way ANOVA followed by a Dunnett's multiple comparison test. ns, no significant difference. The asterisks refer to the level of significance, ^*^p < 0.05; ^**^p < 0.01; ^***^p < 0.001.

To assess the breadth of antibody responses induced by the cHA-based vaccinations, total IgG antibody titers against H1, H2, H18 (group 1), and H3 (group 2) HAs were measured on day 91 by ELISA (Figure 4). The Prime-LAIV-IIV vaccination regimen boosted higher H1-specific IgG titers than the Prime-LAIV-LAIV vaccination group, reaching a GMT of 1:51,200. The LAIV-LAIV vaccination strategy also induced notable H1-specific IgG titers, reaching a GMT of 1:30,444 (Figure 4A). For cross-reactive antibody responses, the Prime-LAIV-LAIV group induced comparable cross-reactive H2-specific IgG titers with the Prime-LAIV-IIV vaccination regimens. Prime-LAIV-LAIV vaccination also induced significantly higher H2-reactive IgG titers than mock immunization (*p* = 0.02). Notably, LAIV-LAIV immunization also induced higher H2-specific IgG antibody titers than the LAIV group (Figure 4B). A similar pattern of antibody responses was observed between H1 and H18 (a more distantly related group 1 HA as compared to H1), albeit H18 titers were lower in all vaccination groups. Again, the Prime-LAIV-IIV regimen induced higher H18-reactive IgG antibodies (GMT of 1:2691) than the other groups (Figure 4C). However, only low antibody titers specific for the group 2 H3 HA were detected in all immunized groups (Figure 4D). Taken together, our results indicated that cHA-based Prime-LAIV-LAIV and/or Prime-LAIV-IIV vaccination approaches boosted HA stalk-specific and broadly cross-reactive IgG antibody responses against group 1 HA (H1, H2, and H18), but did not induce cross-group (group 2, H3) reactive antibodies.

### Sequential Immunization With LAIV-LAIV Confers the Best Protection Against pH1N1 Viral Challenge

**Figure 5:**
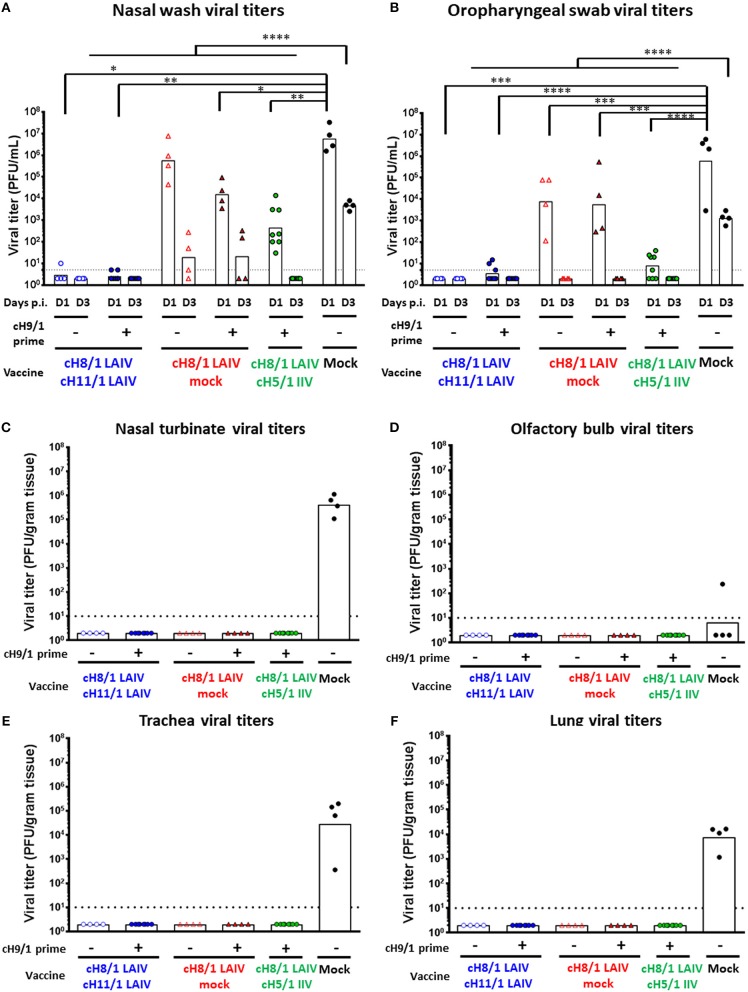
Viral replication post pH1N1 (10^6^ PFU) challenge infection. Viral titers of each vaccinated group or mock animals were measured by plaque assay. LAIV-LAIV vaccinated animals are shown as blue circles. Single dose of LAIV vaccinated animals are shown as red triangles. LAIV followed by AS03-adjuvanted IIV vaccinated animals are shown as green circles. Mock-immunized animals are shown as black circles. Empty symbols denote unprimed. Closed symbols denote B-cH9/1 virus prime immunization. White bars indicate the GMT with individual scatter dot plots. Each point indicates the titer for each individual ferret (*n* = 4 or 8/group). The black dashed line indicates the limit of detection for the assay. **(A)** Nasal wash and **(B)** Oropharyngeal swab viral titers were measured on days 1 and 3 post infection. **(C)** Nasal turbinate, **(D)** olfactory bulb viral titers in the upper respiratory tract, and **(E)** trachea, **(F)** lung (left medial bronchus of the upper lobe) viral titers in the lower respiratory tract were determined on day 4 post challenge. Groups in **(A)** and **(B)** were compared to mock vaccinated animals with one-way ANOVA followed by a Dunnett's multiple comparison test. The asterisks refer to the level of significance. ^*^p < 0.05; ^**^p < 0.01; ^***^p < 0.001; ^****^p < 0.0001.

To evaluate the efficacy of protection elicited by the distinct vaccine regimens, all ferrets were challenged by intranasal infection with 10^6^ PFU of a pH1N1 influenza virus isolate (Cal/09) on day 91 post-prime immunization. Nasal wash and oropharyngeal swab samples were harvested on days 1 and 3 post-challenge to examine the kinetics of virus replication in the upper respiratory tract. All animals were then humanely euthanized on day 4 post-challenge infection for collection of respiratory tract specimens and analysis of viral titers (Figure 1). As expected, the mock-immunized animals had the highest viral titers in nasal wash samples on days 1 and 3 post-pH1N1 virus infection, with GMT of 5.88 × 10^6^ and 4.72 × 10^3^ PFU/mL, respectively. Strikingly, both LAIV-LAIV vaccinated groups significantly suppressed viral replication in the upper respiratory tract on days 1 and 3 post-pH1N1 challenge infection, irrespective of the B-cH9/1 prime immunization. Moreover, the Prime-LAIV-Mock and Prime-LAIV-IIV vaccinated ferrets had decreased viral titers on day 1 post-challenge infection with titers of 1.56 × 10^4^ and 4.5 × 10^2^ PFU/mL, respectively, and almost undetectable viral titers on day 3 post-infection (Figure 5A). Similar trends were also observed for the oropharyngeal swab viral titers, albeit at lower viral titers. Again, animals which received Prime-LAIV-LAIV, LAIV-LAIV, or the Prime-LAIV-IIV vaccination regimen showed greater reduction of oropharyngeal swab viral titers on day 1 post-infection than those animals that received a single dose of LAIV or the mock immunization (Figure 5B). Interestingly, all vaccinated groups were completely protected against viral replication throughout the respiratory tract, whereas the pH1N1 influenza virus replicated efficiently in the nasal turbinate, trachea, and lung of the mock-immunized control ferrets, with GMTs of 4.14 × 10^5^, 2.82 × 10^4^, and 7.6 × 10^3^ PFU/gram tissue, respectively (Figures 5C,E,F). Undetectable levels of viral replication were observed in the olfactory bulb for all immunized animals except for one mock-immunized animal with a titer of 2.4 × 10^2^ PFU/gram tissue (Figure 5D). In summary, we demonstrated that the Prime-LAIV-LAIV and Prime-LAIV-IIV vaccinations induced superior HA stalk-specific antibody responses and provided better protective immunity against pH1N1 virus infection.

### cHA-Based LAIV-LAIV and LAIV-IIV Vaccine Regimens Conferred Protection Against H6N1 Influenza a Virus Challenge Infection

**Figure 6:**
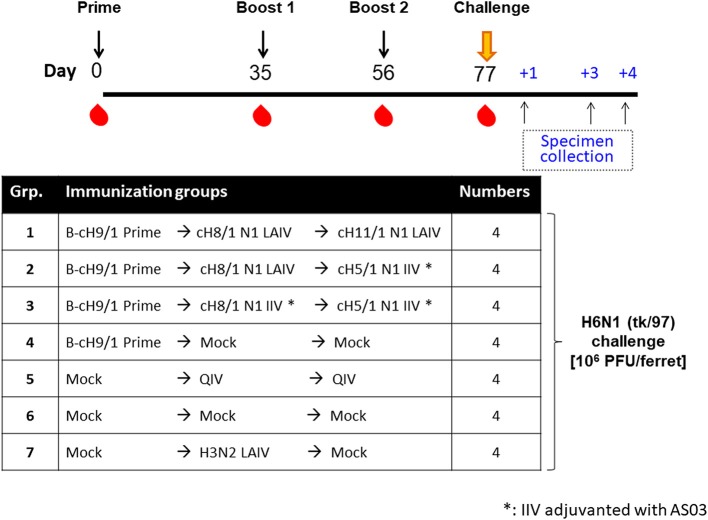
Overview of the H6N1 viral challenge study design. The timeline and experimental groups for the H6N1 challenge study are outlined. All cHA-based vaccinated ferrets were prime immunized with B-cH9/1 virus, followed by booster immunization with cH8/1 LAIV or AS03-adjuvanted cH8/1 IIV on day 35, followed by a second booster immunization with cH11/1 LAIV or AS03-adjuvanted cH5/1 IIV on day 56. In addition, a prime-only, QIV-QIV, H3N2 LAIV, or mock-vaccinated ferrets were included as control groups. All ferrets were challenged with the H6N1 (tk/97) influenza A virus on day 77, and sample specimens were collected on days 1, 3, and 4 post-challenge infection.

**Figure 7:**
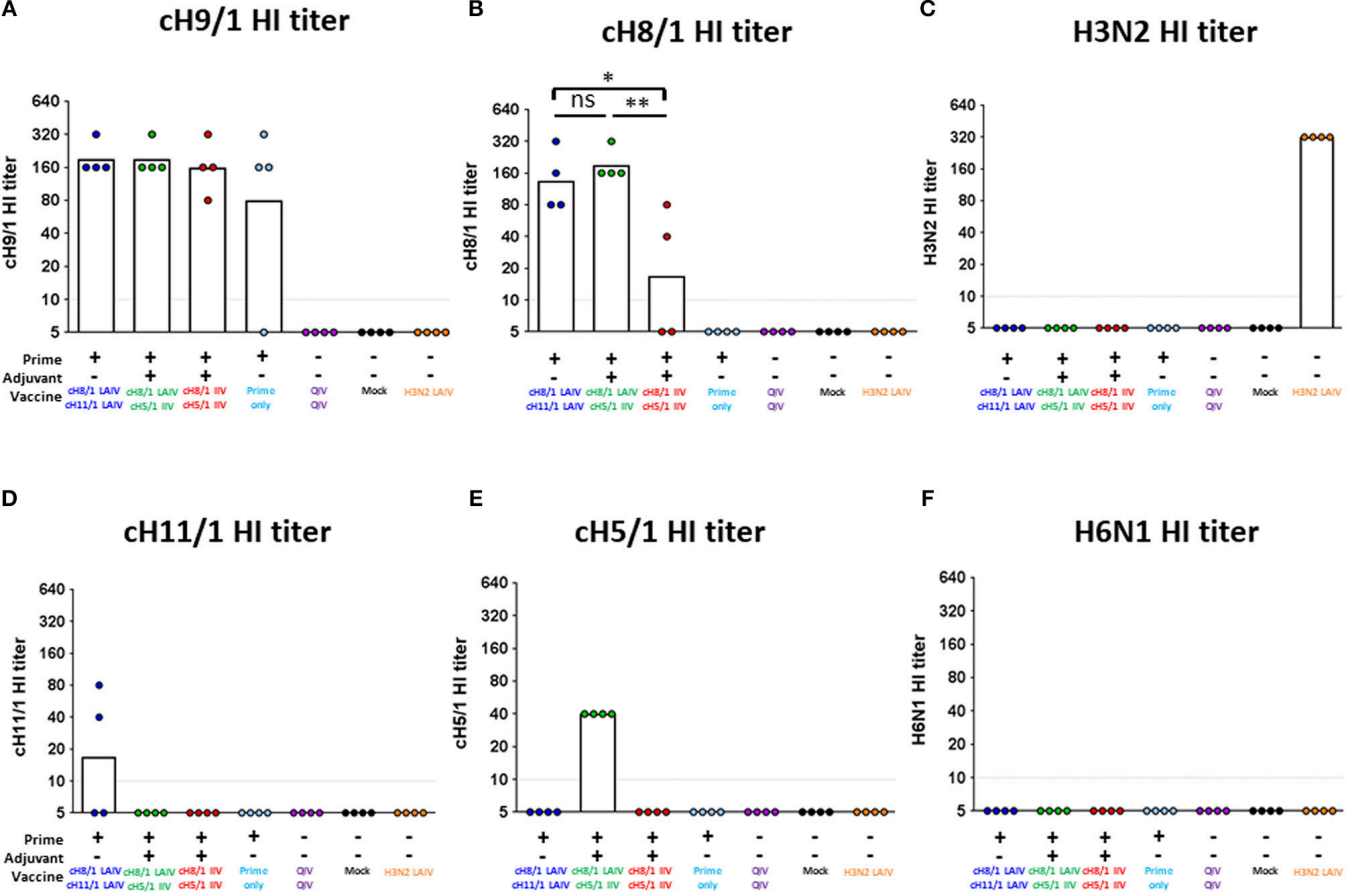
Vaccine-specific antibody titers measured by hemagglutination inhibition assay. Pre-challenge (day 77) hemagglutination inhibition (HI) titers against **(A)** cH9/1, **(B)** cH8/1, **(C)** H3N2, **(D)** cH11/1, **(E)** cH5/1, and **(F)** H6N1 viruses were measured prior to H6N1 influenza A virus challenge infection. Y-axis indicates HI titers against the different virus strains. LAIV-LAIV vaccinated animals are shown in blue. LAIV followed by AS03-adjuvanted IIV vaccinated animals are shown in green. Two doses of AS03-adjuvanted IIV vaccinated animals are shown in red. Prime only, two doses of QIV, and single dose of H3N2 LAIV vaccinated animals are shown in light blue, purple, and orange, respectively. Mock-immunized animals are shown as black. All cHA-vaccinated ferrets were primed with influenza B-cH9/1 virus. White bars indicate the GMT with individual scatter dot plots. Each point indicates the titer for each individual ferret (*n* = 4/group). The black dashed line indicates the limit of detection for the assay. Data were analyzed by one-way ANOVA with Tukey multiple comparisons test. ns, no significant difference. The asterisks refer to the level of significance. ^*^p < 0.05; ^**^p < 0.01.

The results from the first study (pH1N1 influenza virus challenge) suggested that an LAIV component induced robust HA stalk-specific antibody responses and conferred the greatest level of protection against infection by pH1N1 influenza virus. To confirm this observation, the LAIV-LAIV (group 1) and LAIV-IIV (group 2) vaccine regimens were selected for a follow-up vaccination study. Since our previous study demonstrated that IIV-IIV vaccination also elicited high stalk-reactive antibody titers (19), here we further examined whether H1 stalk-specific antibodies were sufficient to prevent infection by a heterosubtypic H6N1 influenza virus. An additional IIV-IIV vaccination group in which cH8/1 was delivered as IIV (Group 3) was included. A control group of ferrets was sequentially immunized with two doses of quadrivalent inactivated influenza virus vaccine (QIV-QIV) to mimic the standard of care for human immunizations (Group 5). Lastly, an A/Ann Arbor/6/1960-based H3N2 LAIV immunization group was included to examine the potential contribution of cellular immune responses toward internal proteins (i.e., NP, M1, NS1, etc.) to protection against influenza virus infection (Group 7). Mock-immunized ferrets received allantoic fluid (Group 6). The detailed vaccination regimens are summarized in Figure 6. Since A/teal/HK/W312/97 (tk/97) H6N1 virus was reported to replicate efficiently in ferrets (34), we selected this avian influenza virus as the challenge virus in this study. This H6N1 (tk/97) virus encodes an H6 (group 1 HA) stalk, which is antigenically distinct from the H1 (also group 1) stalk immunogen included in all vaccines used in this study. Vaccine-induced HI titer, H1 stalk-specific, and the breadth of antibody responses in sera were measured and reported in the Figure 6 and Supplementary Figures. In general, the Prime-LAIV-LAIV and Prime-LAIV-IIV regimens showed similar patterns with Figures 2–4. Notably, the Prime-IIV-IIV regimen also elicited high stalk-reactive IgG and IgA and N1-specific antibodies in sera similar to the results from our previous study (19); on the contrary, the H3N2 LAIV vaccinated animals did not induce substantial H1 stalk-reactive IgG and IgA and N1-specific antibody responses in serum and/or nasal washes (Figures S3, S5). HI titers were also measured on day 77 (pre-challenge). Ferrets immunized with the influenza B-cH9/1 virus developed H9-specific HI titers ranging from 80 to 320, except for one outlier from the prime only group (Figure 7A). All animals which received the cH8/1 LAIV booster immunization and two ferrets which received the cH8/1 IIV booster immunization developed H8-specific HI titers ranging from 40 to 320 (Figure 7B). The animals which received the H3N2 LAIV vaccine also developed H3-specific HI titers of 320 (Figure 7C). Following the second booster immunization with cH11/1 LAIV or cH5/1 IIV, the ferrets developed H11-specific or H5-specific HI titers that ranged from 5 to 80 or 40, respectively (Figures 7D,E). Moreover, all ferrets were also confirmed to be serologically negative against the H6N1 influenza virus used for the challenge infections (Figure 7F). Taken together, our findings indicated that cHA-based Prime-LAIV-IIV and Prime-LAIV-LAIV regimens afforded robust serum and mucosal antibody responses that could provide heterosubtypic immunity.

### cHA-Based LAIV-LAIV and LAIV-IIV Vaccination Regimens Conferred Cross-Protection Against H6N1 Influenza Virus Challenge

**Figure 8:**
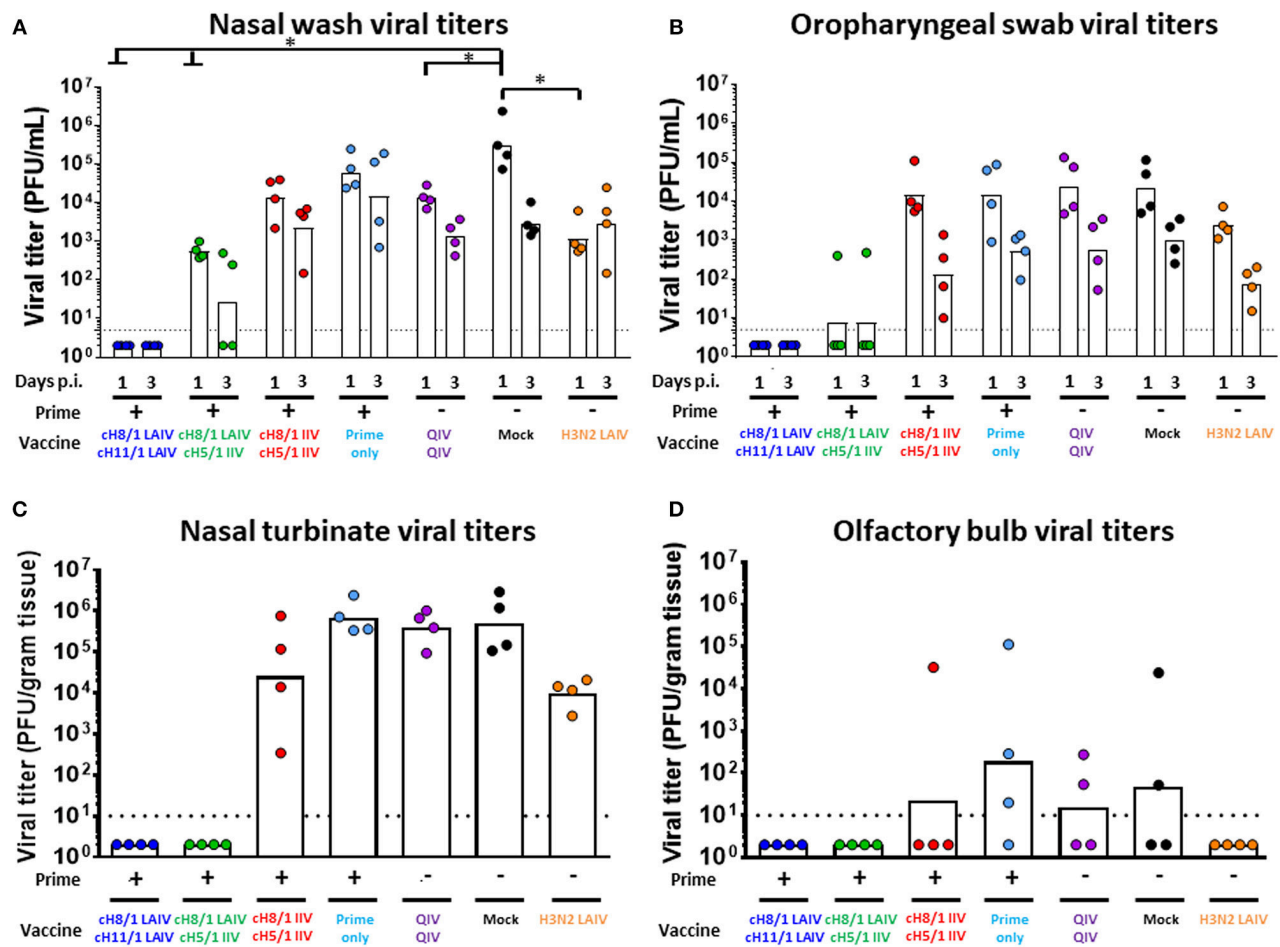
Viral shedding post H6N1 (10^6^ PFU) challenge infection. Viral titers of each vaccinated group or mock animals were measured by plaque assay. LAIV-LAIV vaccinated animals are shown in blue. LAIV followed by AS03-adjuvanted IIV vaccinated animals are shown in green. Two doses of AS03-adjuvanted IIV vaccinated animals are shown in red. Prime only, two doses of QIV, and single dose of H3N2 LAIV vaccinated animals are shown in light blue, purple, and orange, respectively. Mock-immunized animals are shown in black. All cHA-vaccinated ferrets were primed with influenza B-cH9/1 virus. White bars indicate the GMT with individual scatter dot plots. Each point indicates the titer for each individual ferret (*n* = 4/group). The black dashed line indicates the limit of detection for the assay. **(A)** Nasal wash and **(B)** Oropharyngeal swab viral titers were measured on days 1 and 3 post infection. **(C)** Nasal turbinate and **(D)** Olfactory bulb viral titers in the upper respiratory tract were determined on day 4 post challenge. Groups in **(A)** and **(B)** were analyzed by two-way ANOVA followed by a Tukey's multiple comparison test (multiple time points). Groups in **(C)** and **(D)** were analyzed by one-way ANOVA followed by a Tukey's multiple comparison test (single time point). The asterisks refer to the level of significance: ^*^p < 0.05.

Finally, we assessed the protective efficacies of these distinct vaccination regimens against challenge infection by an H6N1 influenza virus. The H6 HA includes an HA stalk that is phylogenetically distinct from the H1 HA stalk but also belongs to the group 1 HAs. Following the sequential immunizations, ferrets were intranasally challenged with 10^6^ PFU of an H6N1 (tk/97) avian influenza virus. Nasal wash and oropharyngeal swab samples were harvested on days 1 and 3 post-challenge infection, and animals were euthanized on day 4 post-challenge for collection of tissue specimens (Figure 6). Body weight changes were also monitored during the infection period. Overall, the Prime-LAIV-LAIV and Prime-LAIV-IIV vaccinated groups did not experience any body weight loss resulting from virus infection as compared to the other immunization groups (Figure S6A). As expected, high viral titers were detected in nasal wash samples of mock-immunized animals, with GMT of 3.15 × 10^5^ PFU/ml. Promisingly, nasal wash viral titers could not be detected from the LAIV-LAIV vaccination group on day 1 post challenge infection. All the other vaccinated groups suppressed virus replication in the upper respiratory tract by 1–2 logs at day 3 post-challenge infection. Our results indicated that the cHA-based Prime-LAIV-IIV vaccination regimen inhibited virus replication as evidenced by low viral GMT of 2.66 × 10^2^ PFU/mL for the day 3 nasal wash samples (Figure 8A). Similar trends in virus titers were also obtained for the oropharyngeal swab samples, but toward lower titers (Figure 8B). Virus replication in the upper and lower respiratory tracts were also evaluated on day 4 post-challenge. Encouragingly, the Prime-LAIV-LAIV and Prime-LAIV-IIV vaccinated ferrets also effectively suppressed virus replication in the nasal cavity (Figure 8C). Similar trends in virus titers was also observed for the olfactory bulb samples, with olfactory bulb virus titers for the Prime-LAIV-LAIV, Prime-LAIV-IIV, and H3N2 LAIV vaccinated ferrets below the limit of detection (Figure 8D). Interestingly, the H3N2 LAIV immunization suppressed virus replication in the upper and lower respiratory tract which was likely due to T cell-mediated immune responses against the internal viral proteins, but independently of HA stalk-specific antibody responses (Figures S3B,C). Unfortunately, the H6N1 (tk/97) virus used in this study did not replicate consistently in the lower respiratory tract (trachea and lung) as virus replication was only detected in a few animals from the prime-only, QIV-QIV, or mock-immunized groups (Figures S6B,C). In summary, the cHA-based Prime-LAIV-LAIV vaccination regimen conferred the best protection against H6N1 virus infection.

### cHA-Based LAIV-LAIV Vaccine Regimens Induced HA Stalk-Specific T Cell Immune Responses

**Figure 9:**
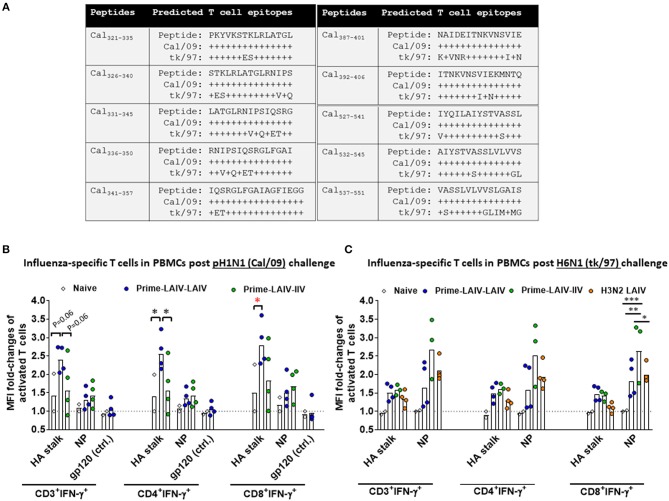
Analysis of antigen-specific T cell responses in PBMCs by flow cytometry. **(A)** Ten peptide sequences corresponding to predicted T cell epitopes within the Cal/09 (H1N1) stalk domain were identified by computational analysis. Alignments of the HA stalk peptides between Cal/09 (H1N1) and tk/97 (H6N1) are summarized. Non-conserved amino acids are indicated. Influenza virus HA stalk-specific, NP-specific and gp-120-specific T cell responses from PBMCs are shown for the Prime-LAIV-LAIV (blue circles), Prime-LAIV-IIV (green circles), or naïve animals (white diamonds) following challenge infection with pH1N1 influenza virus **(B)** or H6N1 influenza virus **(C)**. X-axis indicates influenza HA stalk-specific, NP-specific, and irrelevant peptide gp120-specific CD3^+^IFN-γ^+^, CD4^+^IFN-γ^+^ or CD8^+^IFN-γ^+^ T cells. Fold changes in mean fluorescence intensity (MFI) of IFN-γ expressions over the MFI of naïve animals were calculated and plotted on the y-axis. One sample for a Prime-LAIV-IIV vaccinated ferret in **(C)** was contaminated and was not included in the analysis. Each point indicates the fold-change in MFI of activated T cells for each individual ferret. The black dashed line indicates the averaged baseline responses of naïve animals. Data were analyzed by two-way ANOVA followed by a Tukey's multiple comparison test (different peptides stimulation). The asterisks refer to the level of significance: ^*^p < 0.05; ^**^p < 0.01; ^***^p < 0.001.

In addition to humoral immunity, cellular immune responses may also play a crucial role for cross-protective and long-lasting protective immunity. Therefore, we examined T cell responses elicited by the best cHA-based vaccination regimens (Prime-LAIV-LAIV and Prime-LAIV-IIV) at day 4 post-viral challenge to assess the recall of cellular responses that may have contributed to the observed protection against pH1N1 or H6N1 influenza virus infection. To analyze influenza virus-specific T cell responses to the H1 stalk domain, we designed a panel of peptides based on A/California/04/09 (Cal/09) sequences, which potentially correspond to predicted CD4^+^ and/or CD8^+^ T cell epitopes. Overall, the ten Cal/09 HA stalk-based peptides shared 53–88% identity with the H6N1 (tk/97) strain (Figure 9A). Nucleoprotein (NP)-specific T cell responses were measured following stimulation of peripheral blood mononuclear cells (PBMCs) with a pool of Cal/09 (H1N1) virus NP 15-mer peptides that overlapped by 11 amino acids. The amino acid sequences of the NP encoded by Cal/09 and tk/97 virus strains share 93% identity. CD4^+^ and CD8^+^ IFN-γ^+^ effector T cells in PBMCs or mediastinal lymph nodes (MLNs) were analyzed by flow cytometry after peptide stimulation. An HIV gp120 peptide was included as a control. The gating strategy is diagrammed in Figure S7A. The Prime-LAIV-LAIV vaccination regimen induced higher HA stalk-specific T cell (both CD4^+^IFN-γ^+^ and CD8^+^IFN-γ^+^) responses among PBMCs as compared to the naive immunized group. No apparent differences for NP-specific CD4^+^IFN-γ^+^ and CD8^+^IFN-γ^+^ T cells were observed. Low T cell responses for the naïve group were observed upon stimulation with irrelevant gp 120 peptide (Figure 9B). From the H6N1 challenge study, we observed that the Prime-LAIV-LAIV and Prime-LAIV-IIV vaccinated ferrets induced slightly higher frequency of H1 stalk-specific effector T cells (CD3^+^IFNγ^+^, CD4^+^IFNγ^+^, and CD8^+^IFNγ^+^) than the naïve immunized animals (Figure 9C). The Prime-LAIV-IIV regimen induced more NP-specific CD4^+^IFNγ^+^ and CD8^+^IFNγ^+^ T cell responses than the Prime-LAIV-LAIV or H3N2 LAIV vaccinated group. The H3N2 LAIV vaccinated control group also stimulated NP-specific, but not HA stalk-specific, T cell responses (both CD4^+^ and CD8^+^), which supports the concept that the LAIV backbone induces cell-mediated immunity (Figure 9C). In addition to circulating PBMCs, we also examined whether the H6N1 challenge infection recalled T cell responses within draining lymph nodes. Accordingly, we measured HA stalk-specific and NP-specific T cell responses in mediastinal lymph nodes after 4 days post-H6N1 viral challenge. Based on gating of CD3^+^ T cells, the Prime-LAIV-LAIV vaccinated group induced slightly higher percentages of HA stalk-specific and NP-specific T cells (both CD4^+^IFNγ^+^ and CD8^+^IFNγ^+^) than the Prime-LAIV-IIV, H3N2 LAIV and mock immunized groups (Figures S7B,C).

## Discussion

Targeting the highly conserved HA stalk region (6, 14, 17, 19, 24) of influenza viruses has recently become a promising strategy for the rational design of universal influenza virus vaccines. Our recent studies demonstrated that cHA-based LAIV-IIV vaccination regimens conferred better protection against pH1N1 viral challenge than that provided by sequential immunization with cHA-based IIV-IIV or trivalent influenza vaccine (TIV)-TIV in a preclinical ferret model (18, 19). The aims of this preclinical study were to assess whether HA stalk-specific antibody responses could be further enhanced by sequential immunization with cHA-based LAIV vaccines, and to examine the breadth of protective immunity induced by our sequential immunization regimen. As expected, we confirmed that Prime-LAIV-IIV and Prime-IIV-IIV vaccination regimens induced comparable broadly-reactive and HA stalk-specific humoral immune responses (Figures S3, S4); however, lower stalk-reactive IgG titers in serum and IgA titers in nasal wash samples were observed for the Prime-IIV-IIV vaccination regimens than in our previous study (Figure S3) (19), which may be attributed to the genetic diversity of the outbred ferret model. The results from the Prime-LAIV-IIV regimens also indicated that immunization with the cH9/1 influenza virus primed strong H1 stalk-specific and broad antibody responses (Figures 3, 4). Recently, Isakova-Sivak et al. reported that two doses of cHA-based LAIV vaccines (Len/57-based) induced greater broadly-reactive and HA stalk-reactive IgG antibodies when compared to immunization with Len/57-based LAIV expressing wild-type HAs in mice (15). Our data echo their results. Additionally, Hoft et al. demonstrated that children immunized with a seasonal LAIV vaccine (FluMist) induced stronger influenza virus-specific secretory IgA responses than those immunized by IIV (31, 35). Our results also showed that ferrets immunized with cHA-based Prime-LAIV-LAIV vaccines induced superior IgA titers in nasal washes at 3 days post pH1N1 or H6N1 challenge than all the other immunized groups (Figures S2B, S3D). With respect to the protective immunity elicited by our Prime-LAIV-LAIV vaccination approach, our findings suggest that serum and mucosal antibody responses in combination with cell mediated immunity are required to provide complete protection against influenza virus infection. The quality and level of stalk-specific antibodies elicited by the vaccine may also determine the breadth and duration of protection against distinct drifted or pandemic strains of influenza virus. A recent study demonstrated in the mouse model that in the absence of immunodominant B cells, plasma B cells recognizing subdominant B cell epitopes may selectively expand, increase germinal center (GC) reactions, and differentiate into long-lived memory B cells (36). A recent study also demonstrated that immunization with an H5N1 LAIV vaccine primed GC B cell reactions and stimulated the generation of plasmablast and memory B cells in MLN, and that booster immunization with inactivated subunit vaccine recalled these H5-specific B cell responses in axillary lymph nodes, spleen and peripheral blood in African green monkeys (37). Although the precise protective mechanisms underlying the humoral immunity induced by our vaccination regimen are still unclear, recent studies have highlighted the contribution of Fc-dependent mechanisms to protection by broadly-reactive neutralizing antibodies (38–40). It will be interesting to further dissect the development of long-lived B and T cell responses induced by sequential immunization with our cHA-based vaccines. Along these lines, immunological reagents and assays are being developed for the ferret model (41), which is an animal model of influenza that was highlighted in the National Institute of Allergy and Infectious Diseases (NIAID)'s strategic plan for the development of universal influenza vaccines (7).

As expected, vaccination with the H3N2 LAIV vaccine did not induce H1 stalk-specific antibody responses (Figure S3); however, H3N2 LAIV vaccination suppressed viral replication in the upper respiratory tract following H6N1 virus challenge (Figure 8), which was likely due to cell mediated immune responses against the conserved internal proteins. Contrary to another recent report (34), we did not detect replication of the tk/97 H6N1 virus in the lower respiratory tract despite detecting viral replication in the nasal turbinates (Figure 8C, Figures S6B,C). One possible explanation for this discrepancy is differences in methods to quantify influenza virus titers. Herein, virus titers were quantified by traditional plaque assay on MDCK cells, whereas in the previous report virus titers were quantified by 50% egg infectious dose (EID_50_) per gram of tissue. Of note, the A/teal/HK/W312/97 (tk/97) H6N1 influenza virus used for the second vaccination/challenge experiment encodes a glutamate residue at position 627 of the PB2 which is a host determinant for influenza virus replication in avian species (42).

Recent findings have emphasized that cellular immune responses contribute to protection against influenza virus infection. Consistently, two recent clinical studies demonstrated that vaccinees who received LAIV vaccines significantly increased T cell responses (28, 29, 31). Influenza virus-specific memory CD8^+^ T cells in the airway (43) and lung-resident CD4^+^ and CD8^+^ memory T cells (Trm) (44) appear critical for cross-protection against influenza virus infection. In addition, a recently identified CD4^+^ T cell subset, the follicular helper T cells (Tfh), has been reported to coordinate GC reactions to stimulate the expansion of plasmablast and memory B cells, thus inducing high quality influenza virus-specific antibody responses (45, 46). To what extent our universal influenza virus vaccine approach stimulates such T cell responses will be the subject of future immunological studies. Although our findings demonstrated that our cHA-based Prime-LAIV-LAIV and Prime-LAIV-IIV vaccination regimens induced stalk-specific humoral immunity and cellular immunity (Figure 9, Figure S7) which conferred cross-protection against influenza virus infection, the results obtained with this animal model of influenza, while encouraging, have yet to provide a good correlation between any immune readouts and protection. Interestingly, fewer peripheral Cal/09 HA- and/or NP-specific T cells were recalled in the Prime-LAIV-LAIV immunized group at day 4 post-H6N1 challenge infection. This may be accounted by the low homology between the Cal/09 (H1N1) versus tk/97 (H6N1) hemagglutinin stalk peptides (Figure 9A).

The Advisory Committee on Immunization Practices (ACIP) of the Centers for Disease Control and Prevention (CDC) recently announced the re-inclusion of LAIV vaccines into the list of recommended vaccines for the 2018–2019 influenza season (47). This change in clinical practice by the ACIP supports our efforts to translate our universal influenza virus vaccine that includes an LAIV component to clinical trials, especially for the pediatric and adult populations. In particular, incorporation of our universal vaccine approach into childhood immunization practice may confer “immunologic imprinting” of HA stalk immunity that provides more desirable immunologic responses to future immunization with influenza virus vaccines and provide broader protection against influenza virus infection (48). The National Institute of Allergy and Infectious Diseases (NIAID) recently published a position statement in which they outlined the critical elements of a universal influenza virus vaccine: (i) provide ≥ 75% protection against symptomatic disease caused by infection with group 1 and group 2 influenza A viruses, and (ii) afford ≥ 12 months protection in all populations (3). In summary, the results of this preclinical study suggest that, of the sequential immunization approaches examined, the Prime-LAIV-LAIV vaccination approach is the most promising vaccination regimen to confer protection against infection by influenza A viruses encoding a group 1-type hemagglutinin. However, the immune response following the tested vaccine regimens in ferrets might be substantially different than the immune response in humans with pre-existing immunity. It will therefore be important to compare these approaches in clinical trials before final conclusions are drawn. Although, herein, we have not addressed all criteria noted above, the current findings encourage the development of universal influenza virus vaccine components that target group 2 influenza A viruses and influenza B viruses. The findings reported herein are consistent with our previous findings (18, 19) and collectively demonstrate that influenza virus vaccines that focus immune responses on the HA stalk domain are a viable approach to a universal influenza virus vaccine. Ideally, our vaccine candidates will be able to induce not only stalk-reactive antibodies against all influenza subtypes, but also influenza virus-specific cellular immune responses.

## Materials and Methods

### Cells, Viruses, and Proteins

Madin-Darby Canine Kidney (MDCK) cells used for plaque assays were maintained in Minimum Essential Media supplemented with 10% fetal bovine serum (FBS, Hyclone) and 100 U/mL penicillin plus 100 μg/mL streptomycin (1x P/S). Virus stocks were grown in 8-day-old specific pathogen-free embryonated chicken eggs (Charles River Laboratories) at 37°C for 2 days or at 33°C for 3 days, and were then chilled down to 4°C overnight. Allantoic fluid was harvested, centrifuged at 290 × g for 5 min, and titrated by plaque assays as previous study described (19). Plaques were visualized by immunostaining or crystal violet staining. Recombinant proteins utilized in this study consist of chimeric H6/1 [containing H6 head domain from A/mallard/Sweden/81/02 combined with an H1 stalk domain of A/California/04/09 (Cal/09)], H1 (Cal/09), H2 (A/mallard/Netherlands/5/99), H3 (A/Hong Kong/4801/14), H6 (A/mallard/Sweden/81/02), H18 (A/bat/Peru/33/10), and N1 (Cal/09). Recombinant proteins were expressed in High Five cells grown in serum free SFX medium (HyClone) using the baculovirus expression system (49).

### cHA-Based Universal Vaccine Preparations

The IIV split virus vaccines, produced in 8–10 day-old chicken embryonic eggs as previously reported for the manufacture of pandemic H5N1 split virus vaccine, were provided by GlaxoSmithKline (GSK). The cH5/1_Cal09_N1_Cal09_ (cH5/1) IIV is composed of an HA head domain of A/Vietnam/1203/04 (H5N1), an H1 stalk domain and N1 NA from the Cal/09 strain while the internal genes were derived from the H1N1 A/Puerto Rico/8/34 (PR8) virus. The cH8/1_Cal09_N1_Cal09_ (cH8/1) IIV consists of an HA head domain from A/mallard/Sweden/24/02 (H8N4) while the remaining genes as well as the stalk domain are the same as for the cH5/1 IIV. All IIV vaccines used in this preclinical study were adjuvanted with Adjuvant System 03 (AS03) in the same formulation as for our previous study (19, 32), and which has been licensed for use in humans for pandemic influenza virus vaccines (50). The backbone of the cH8/1 and cH11/1_Cal09_N1_Cal09_ (cH11/1) LAIVs are based on the Leningrad vaccine strain (A/Leningrad/134/17/57, Len/57), which has been licensed and successfully used in humans (51). The H3N2 LAIV virus used in this study was based on the LAIV strain, A/Ann Arbor/6/60, which has been used to produce FluMist LAIV vaccines. The H3N2 LAIV expresses the H3 HA and N2 NA from the H3N2 influenza virus, A/Wyoming/03/03. pDZ plasmids for the virus rescue were cloned at the Icahn School of Medicine at Mount Sinai (ISMMS) as our previous study described (19). cH8/1 LAIV seed virus was rescued by reverse genetics at the US Centers for Disease Control and Prevention (52) and produced at ISMMS. The composition of recombinant B cH9/1 virus (a B/Yamagata/16/88 virus that expresses a cH9/1 HA) was as previously described (20).

### Ferret Immunization and Challenge

Outbred 4 to 5-month-old castrated male Fitch ferrets were confirmed to be seronegative for circulating H1N1, H3N2, and B influenza viruses prior to purchase from Marshall BioResources (North Rose, NY). All animal experiments were performed according to a protocol approved by the Institutional Animal Care and Use Committee (IACUC) of the ISMMS. Ferrets were randomly assigned to the different treatment groups. The number of ferrets (*n* = 4 or 8) included in each experimental group is indicated in the figure legends. Figures 1, 6 summarize the prime-boost immunization strategies performed in this preclinical study. To establish baseline, group 1 HA stalk-specific immunity, naive ferrets in the cHA-based vaccination groups were prime immunized by intranasal administration of 10^6^ PFU of B-cH9/1 virus as in our previous approach (19, 20, 53). Importantly, this priming strategy prevents the establishment of immunity against influenza A virus internal proteins and does not interfere with a later pH1N1 or H6N1 viral challenge. The intervals of prime-boost vaccinations and detailed immunization regimens were indicated in Figures 1, 6 summarize. The dosage of either cH8/1 or cH11/1 LAIV used in this study was 1 × 10^7^ PFU per animal. Some groups of ferrets were intramuscularly boosted with 0.5 mL dose of AS03 adjuvanted cH5/1 IIV. The serum was separately collected at the indicated time points. Notably, the H6N1 challenge study focused on the best-in-class vaccination approaches (LAIV-LAIV and LAIV-IIV) for a more stringent evaluation of the protective immune responses elicited by these cHA-based vaccines. A “standard of care” group of ferrets which received two human doses of QIV (QIV-QIV Fluarix, GSK) and a single dose of H3N2 LAIV vaccination group were also included. The challenge infection dose of either Cal/09 pH1N1 virus or A/teal/HK/W312/97 (tk/97) H6N1 virus was 10^6^ PFU/per ferret in 1 mL of PBS. For evaluation of vaccine effectiveness from both challenge studies, nasal wash and oropharyngeal swab samples were collected from anesthetized ferrets at day 1 and 3 post-challenge infection. At day 4 post-challenge infection, anesthetized ferrets were euthanized by exsanguination followed by intracardiac injection of Sleepaway euthanasia solution (Fort Dodge, Sodium Pentobarbital). Tissue specimens (olfactory bulbs, nasal turbinates, trachea, and lung) were collected from each individual ferret to quantify viral titers by plaque assays.

### Enzyme-Linked Immunosorbent Assay (ELISA)

HA-specific antibodies in serum and nasal wash samples, and N1-specific antibodies in serum were measured by ELISA with the above-mentioned recombinant proteins. The ELISA was conducted as described in our previous study (19). In brief, for serum IgG, each well of 96-well plate was coated with 0.1 μg of antigen in sodium bicarbonate buffer and incubated overnight at 4°C. Plates were washed three times with PBS containing 0.1% Tween 20 (PBST) and blocked with 220 μl of blocking solution (PBST containing 3% goat serum (Gibco) and 0.5% milk powder) for 1 h at room temperature (RT). Serum samples were 2-fold serially diluted in blocking solution (starting dilution from 1:100) and added into plates and incubated for 2 h at RT. Next, plates were washed three times with PBST and horseradish peroxidase (HRP)-labeled anti-ferret IgG (Alpha Diagnostic International) at a dilution of 1:5000 was added to each well and incubated for 1 h at RT, followed by four times PBST washes and developed by SigmaFast OPD (o-phenylenediamine dihydrochloride). After 10 min, the reaction was stopped by addition of 3 M hydrochloric acid and plates were read at a wavelength of 490 nm in a plate reader (Biotek Synergy H1). The average plus three standard deviations of blank wells was calculated as a cut-off endpoint value. The same procedure was used to measure IgA titers in nasal washes and serum. However, a starting dilution of 1:2 was used for the nasal wash samples. HRP-labeled anti-ferret IgA antibody (Novus Biologicals) was used at a 1:3000 concentration as secondary antibody.

### Enzyme-Linked Lectin Assay (ELLA)

The inhibition of N1NA enzymatic activity in serum was measured by ELLA. To determine the optimal concentration of virus used for neuraminidase inhibition (NI) assay, the NA activity induced by PR8 x H7N1 virus (H7HA from H7HA from dk/mallard/Alberta/24/2001 (H7N1) and N1 NA from Cal/09 strain) was first examined as our previous study described (49, 54). Briefly, each well of 96-well plate was coated with fetuin (Sigma) (150 μl at 50 μg/mL) in sodium bicarbonate buffer and incubated overnight at 4°C. Plates were washed once with PBST and blocked with 200 μl of blocking buffer (PBS containing 1% BSA) for 1 h at RT. While plates were being blocked, virus stock was 2-fold serially diluted in blocking buffer (starting dilution from 1:5). After blocking for 1 h, 100 μl amount of the diluted virus was transferred into fetuin-coated plates and incubated for 2 h at 37°C. Next, after 3 times PBST washes, HRP-conjugated peanut agglutinin (PNA, from *Arachis hypogaea*, Sigma) (100 μl at 5 μg/ml) were added to the plates and held for another 2 h incubation at RT. The supernatants were aspirated, washed three times by PBST and developed by 3, 3′, 5, 5′ tetramethyl benzidine (TMB) substrate (Biolegend). After 15 min, the reaction was stopped by addition of 3 M phosphoric acid and plates were read at a wavelength of 410 nm in a plate reader (Biotek). 10^5^ PFU of virus (in this case, approximately half the maximal OD reading value) was used for subsequent NI assays. For NI assay, ELISA plates were coated and blocked in a same manner used for the NA assay. While plates were blocking, ferret serum samples (from the pre-challenge time point) were 2-fold serially diluted in a separate 96-well plates using blocking buffer, starting with a 1:50 dilution, at a final volume of 125 μl. One hundred and twenty five microliter of virus stock (at a concentration of 10^5^ PFU) was added on top of the diluted serum and briefly tapped for mixture, and incubated at 37°C for 1 h. Two hundred microliter of the virus/serum mixture were transferred in parallel to the fetuin-coated plates, after which the plates were incubated at 37°C for 2 h. The rest of the NI assay protocol was identical to that of the NA assay. The readouts obtained from the plate reader were plotted in Prism 7.0 software, normalized by the average of the negative control (defined as 0%) and the average of positive control (virus only, defined as 100%). Percent inhibition was calculated by subtracting the NA activity from 100. The corresponding 50% inhibitory concentrations (IC_50_s) were defined as the reciprocal serum dilutions inhibiting 50% of viral NA enzymatic activity.

### Hemagglutination Inhibition (HAI) Assays

The HAI assays were performed as described in our previous study (19, 55). In brief, the sera were treated with *receptor-destroying enzyme* (RDE) (*Denka Seiken*) for 16–18 h at 37°C, followed by addition of 2.5% sodium citrate for 30 min at 56°C to block non-specific sialic acid binding. PBS was then added to each sample which resulted in an assay starting dilution of 1:10. Working stocks for each influenza virus strain were prepared by diluting the virus stock to a final HA titer of 8 HA units/50 μL in FTA Hemagglutination Buffer (Phosphate Buffered Saline, pH. 7.2; BD BBL). Twenty five microliter of pre-diluted RDE-treated sera were then mixed with 25 μL of the working stock of each influenza virus strain at 96 V-well microtiter plates and incubated for 30 min at RT to allow any HA-specific antibodies present in the serum to neutralize the influenza virus. Next, 50 μL of a 0.5% suspension of turkey red blood cells was added and incubated for 45 min at 4°C. The HI titer was defined as the reciprocal of the highest dilution of serum that inhibited red blood cell hemagglutination by influenza virus.

### Isolation of PBMCs and Preparation of Single-Cell Suspensions From Ferret Tissues

Peripheral whole blood samples were obtained from anesthetized ferrets by intracardiac puncture and collected in Vacutainer EDTA (K2) tubes on the last day of the virus challenge experiment. Whole blood was gently layered onto HistoPaque-1077 (Sigma-Aldrich) in a 1:1 ratio and immediately centrifuged at 400x *g* for 30 min at RT with the lowest accelerating and brake off for descending. The buffy coat containing the peripheral blood mononuclear cells (PBMC) was collected and washed with enriched RPMI (eRPMI) media (RPMI1640 media containing 5% FBS and 10 mM HEPES [4-(2-hydroxyethyl)-1-piperazineethanesulfonic acid)]. Contaminating red blood cells (RBC) present in the single-cell suspensions were lysed with RBC lysis buffer (Affymetrix eBioscience). Mediastinal lymph nodes (MLNs) were excised, dissociated with digestion media [eRPMI media containing 30 μg/mL of DNase I (grade II, Sigma-Aldrich) and 1 mg/mL of Type IV collagenase (Thermo Fisher)] for 30 min at 37°C. Next, the enzyme digested-lymph nodes were mechanically rinsed and passed through 70 μm nylon cell strainers (BD falcon). Contaminating RBCs were lysed and washed out prior to cryopreservation. Purified single-cell suspensions of PBMC and MLN leukocytes were immediately cryopreserved following isolation from tissues for flow cytometric analyses.

### Synthetic Peptide Panels and Pool Peptide Libraries

Predicted HA stalk-specific peptides were designed based on potential T cell epitopes which have been posted on IEDB database (http://www.iedb.org/) and examined in the cellular immune responses of mice or humans to influenza virus (56–59). Briefly, we identified predicted epitopes on influenza virus HA region by searching the IEDB database which included 736 records with supporting systematic analysis (59). We then compared these predicted epitopes with previously published epitopes from human (56) and/or mouse models (57, 58) of infection. Based on the most conserved overlapped sequences, we selected specific epitope sequences for peptide synthesis. Herein, we generated a panel of ten peptides to span the major predicted epitopes in the HA stalk region of the Cal/09 (H1N1) strain (*GenScript peptide services)*. Each peptide was composed of 15 to 17-mers that overlapped by 10 amino acids (aa) to cover the predicted region as shown in Figure 9A. Each peptide was reconstituted as 1 mg/mL stock using a dimethyl sulfoxide. Each peptide was freshly pooled in complete RPMI media (cRPMI, RPMI 1640, 1X P/S, 10% heat-inactivated, certified FBS (Gibco), 10 mM HEPES, 1 mM sodium pyruvate, 1x non- essential amino acid and 50 μM β-mercaptoethanol) at a final working concentration of 4 μg/ml prior to use. Moreover, a pool of Cal/09 (H1N1) virus nucleoprotein (NP) 15-mer peptides that overlapped by 11 aa was included to examine NP-specific T cell responses (Miltenyi Biotec). An irrelevant peptide from HIV-1 envelop protein gp120 (aa_283−297_) (strain BRU, Bachem Inc) was also included as a negative control. The amino acid sequences for NP encoded by Cal/09 and tk/97 were retrieved from GenBank (accession numbers FJ966083 and AF250480, respectively).

### Flow Cytometry Analyses

We phenotyped the T cell responses following the procedure described in our previous report (60, 61). In brief, single-cell suspensions of PBMCs or MLN leukocytes were thawed and resuspended in cRPMI media containing 10 μg/mL of the protein-transport inhibitor Brefeldin A from *Penicillium brefeldianum* (Sigma-Aldrich). Cells were, respectively stimulated with 0.1 μg of pooled NP peptides, irrelevant gp120 peptide, or 0.1 μg for each HA stalk peptide overnight. The next day, the cells were washed twice using flow cytometry staining buffer containing 2% FBS and 0.01% sodium azide in PBS) and centrifugation at 450x *g* at 4°C. Cells were first stained with a viability dye, LIVE/DEAD Aqua Fixable dead-cell stain kit (Thermo Fisher), according to the manufacturer's instruction, followed by surface staining with specific T cell markers [CD4 (clone 02, Sino Biological Inc) and CD8 (OK-T8, Affymetrix eBioscience)] for 30 min at 4°C. Then, cells were washed, fixed by intracellular Fixation buffer for 20 min at 4°C, and permeabilized using permeabilization buffer (Affymetrix eBioscience). Next, the cells were intracellular stained with anti-CD3 (CD3-12, Abcam) and anti-biotin conjugated IFN-γ (CC302, LifeSpan BioSciences) antibodies for 30 min at RT. Antibodies to CD4, CD8, and CD3 were directly conjugated to distinct fluorochromes to distinguish T cell subsets. The anti-biotin IFNγ antibody was detected by a secondary antibody (anti-Strepavidin-APC, Affymetrix eBioscience). After the T cell staining, the cells were washed twice and resuspended in flow cytometry staining buffer for data acquisition using a BD 13-colors LSR-II instrument with 488-, 633-, and 405-nm lasers and FACS Diva software. All data files were analyzed using FCS Express software, version 6.04.0034.

### Statistical Analysis

One-way or two-way ANOVA were, respectively used for comparisons of more than two groups with single or multiple time points, followed by a Tukey's or Dunnett's post-test to adjust for multiple comparisons. All statistical analyses were performed in GraphPad Prism 7.0. The asterisks shown in the figures refer to the level of significance, ^*^p ≤ 0.05; ^**^p ≤ 0.01; ^***^p ≤ 0.001; ^****^p ≤ 0.0001. Serum analysis and viral titer quantification were analyzed in an un-blinded manner.

## Ethics Statement

All animal experiments were performed according to a protocol approved by the Institutional Animal Care and Use Committee (IACUC) of the Icahn School of Medicine at Mount Sinai (ISMMS).

## Author Contributions

W-CL, RN, DS, FK, and RA: performed experiments; W-CL, AS, RN, FB-S, AG-S, PP, FK, and RA: designed and coordinated experiments; W-CL and RA: analyzed data and wrote the manuscript. All authors reviewed and approved the final version of the manuscript.

### Conflict of Interest Statement

AG-S, FK, and PP are inventors in patent applications filed and owned by the Icahn School of Medicine at Mount Sinai regarding influenza virus vaccines. The remaining authors declare that the research was conducted in the absence of any commercial or financial relationships that could be construed as a potential conflict of interest.
