# Supplementary material for: Sequential Immunization With Live-Attenuated Chimeric Hemagglutinin-Based Vaccines Confers Heterosubtypic Immunity Against Influenza A Viruses in a Preclinical Ferret Model
Source: Front Immunol. 2019 Apr 10;10:756. doi: 10.3389/fimmu.2019.00756 (PMC6499175; doi:10.3389/fimmu.2019.00756)
Supplement: Supplementary file 1 [file Data_Sheet_1.pdf]

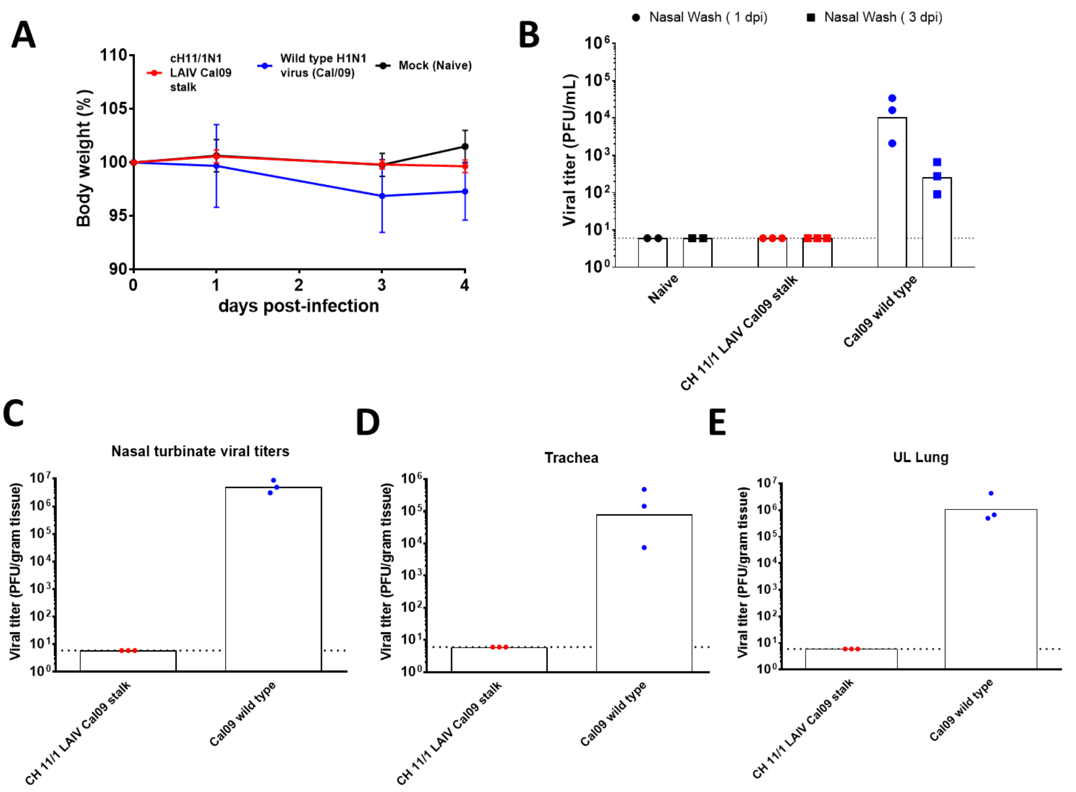

FIG. S1 Pathotyping of cH11/1 LAIV vaccine stocks

(A) Naive ferrets were infected with 10^6 PFU of either LAIV or wild-type H1N1 (Cal/09) virus, and then body weight changes were monitored daily from day 0 to day 4. The mean ± SEM for each group is plotted. (B-E) Influenza virus titers were measured by plaque assay following infection of ferrets (n=3) with one of the following vaccine strains: cH11/1 LAIV Cal09 stalk (red symbols) or wild type Cal/09 (blue symbols). Naïve (mock-immunized) animals are represented by black symbols. The geometric means of the virus titers are plotted with each point indicating the titer for each individual ferret (n = 2 or 3 /group). The black dashed line indicates the limit of detection for the assay. (B) Nasal wash and oropharyngeal swab viral titers were measured on days 1 and 3 post infection. (C) Nasal turbinate, (D) trachea, and (E) lung viral titers were determined on day 4 post infection.

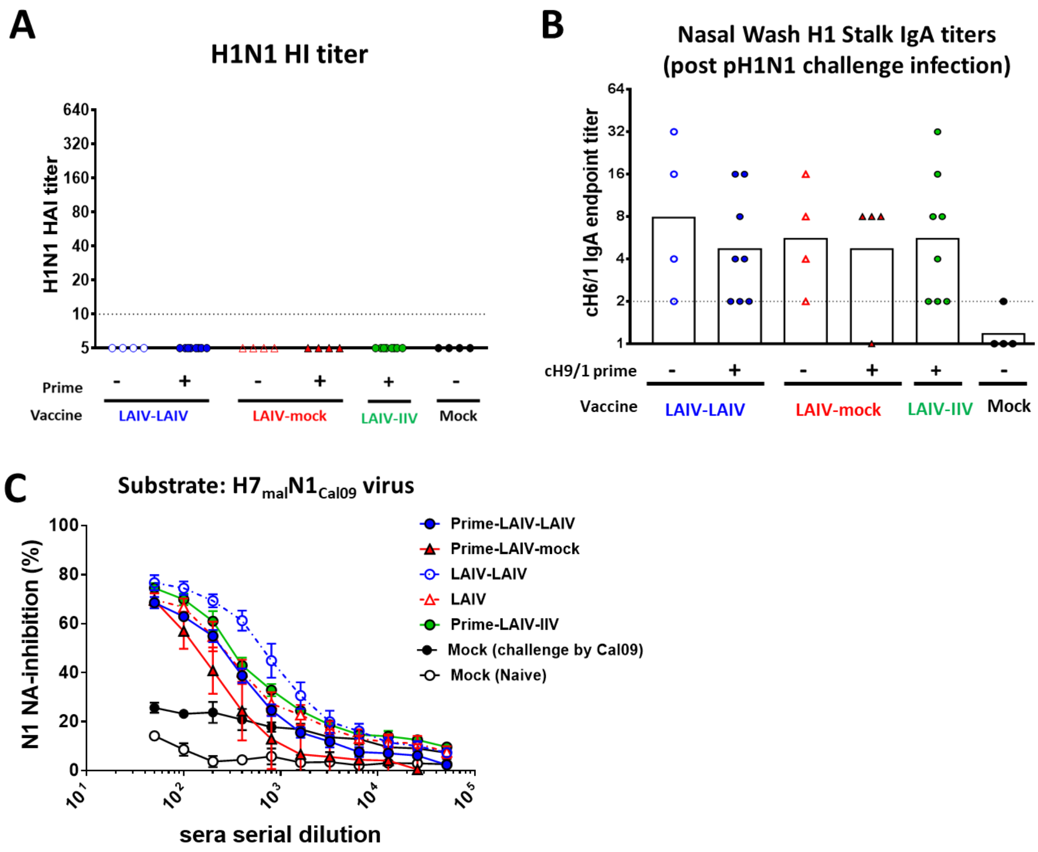

FIG. S2 Serum HA-specific antibody titers and NA-inhibition activity of antibodies prior to pH1N1 challenge

Hemagglutination inhibition (HI) titers against the H1N1 virus (A) and HA stalk-specific IgA titers against cH6/1 recombinant protein (B) was measured prior to pH1N1 influenza A virus challenge (day 91). White bars indicate the GMT with individual scatter dot plots. Each point indicates the titer for each individual ferret (n = 4 or 8/group). The black dashed line indicates the limit of detection for the assay. (C) A fetuin-based enzyme-linked lectin assay was used to measure NA-inhibiting-antibody titers as evidenced by reductions in neuraminidase activity of the H7N1 virus. The NA enzymatic activity curves were normalized based on the negative and positive controls, and NA-inhibition curves were plotted, and the reduction percentages of NA enzymatic activity indicated on the y-axis.

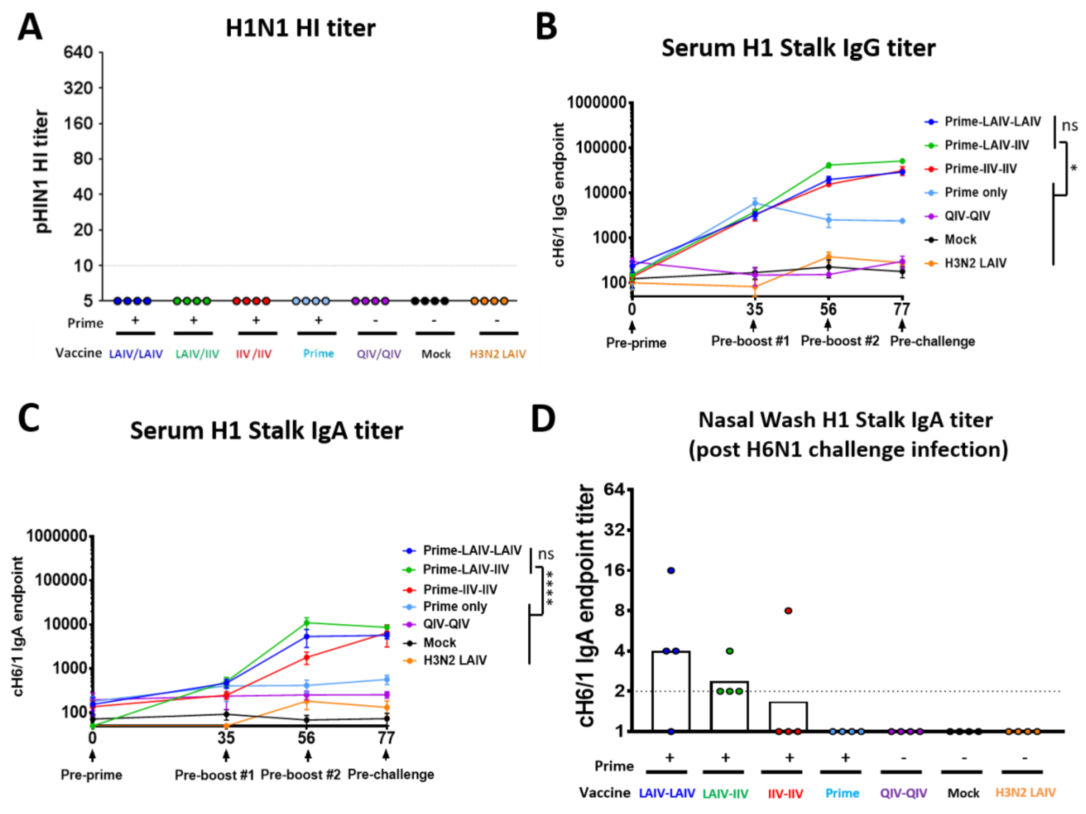

FIG. S3 HA head-specific and stalk-specific antibody titers measured by ELISA.

(A) HI titers against the H1N1 virus was measured prior to H6N1 influenza A virus challenge (day 77). As expected, all vaccinated animals were H1N1-seronegative. The IgG antibody responses in serum were measured on days 0, 35, 56 and 77 (x-axis). (B) H1 stalk serum IgG and (C) H1 stalk serum IgA endpoint titers were measured at the indicated time points post-immunization. (D) H1 stalk nasal wash IgA titers were measured on day 3 post H6N1 challenge infection. Each point indicates the GMT for each individual animal (n=4/group). The white bars indicate the averaged endpoint titer of each experimental group. The black dashed line indicates the limit of detection for the assay. Data in (B, C) were analyzed by two-way ANOVA followed by a Tukey's multiple comparison test (multiple time points). Data in (D) were analyzed by a one-way ANOVA followed by a Tukey's multiple comparison test (single time point). ns: no significant difference. The asterisks refer to the level of significant difference among vaccinated groups at day 77 pre-challenge time point: *: $p<0.05$; ****: $p<0.0001$.

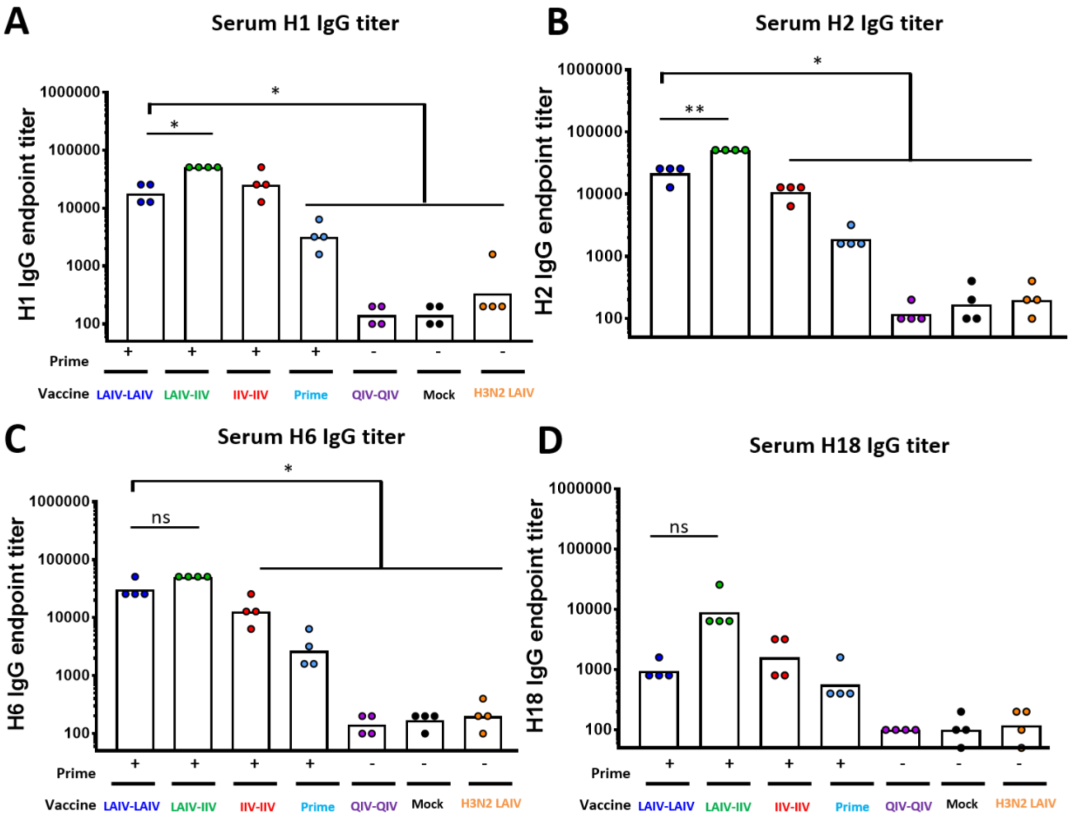

FIG. S4 Breadth of antibody responses measured by ELISA. Serum IgG endpoint titers (y-axis) against (A) H1, (B) H2, (C) H6, and (D) H18 viruses were measured prior to H6N1 challenge infection (day 77). LAIV-LAIV vaccinated animals are shown in blue. LAIV followed by AS03-adjuvanted IIV vaccinated animals are shown in green. Two doses of AS03-adjuvanted IIV vaccinated animals are shown in red. Prime only, two doses of QIV, and single dose of H3N2 LAIV vaccinated animals are shown in light blue, purple, and orange, respectively. Mock-immunized animals are shown in black. All cHA-vaccinated ferrets were primed with influenza B-cH9/1 virus. White bars indicate the GMT with individual scatter dot plots. Each point indicates the endpoint titer for each individual ferret (n= 4/group). Data were compared to Prime-LAIV-LAIV vaccinated animals with one-way ANOVA followed by a Dunnett's multiple comparison test. ns: no significant difference. The asterisks refer to the level of significance: *: p<0.05; **: p<0.01.

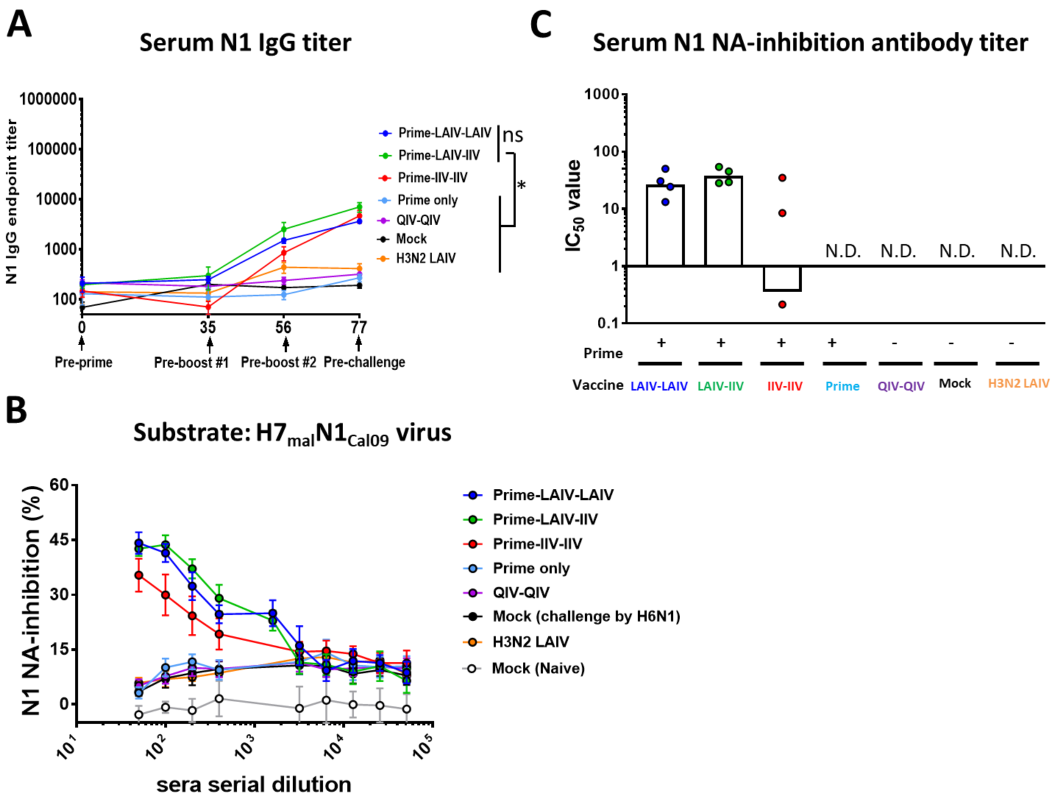

FIG. S5 Anti-N1 NA total IgG titers and anti-N1NA inhibition antibody responses

(A) N1 serum total IgG titer against N1 (Cal/09) protein were measured on days 0, 35, 56 and/or 77 by ELISA and are plotted on the y-axis. (B-C) Fetuin-based enzyme-linked lectin assays were used to measure NA-inhibiting-antibody titers as evidenced by reduction of neuraminidase activity of the H7N1 virus. (B) The percentage of inhibition of neuraminidase activity is graphed as line curves. (C) Corresponding IC_{50} values were determined as the 50% reduction in the NA enzymatic activities of the influenza virus strain, $H7_{mal}N1_{Cal09}$. Each point indicates the IC_{50} value for each individual animal (n = 4/ group). The white bars indicate the GMT of each experimental group. The endpoint titers (A-B) were analyzed by two-way ANOVA followed by a Tukey's multiple comparison test (multiple time points). Data in (B-C) were compared to mock vaccinated animals with one-way ANOVA followed by a Dunnett's multiple comparison test (single time point). ns: no significant difference. The asterisks refer to the level of significance: *: p<0.05; ****: p<0.0001.

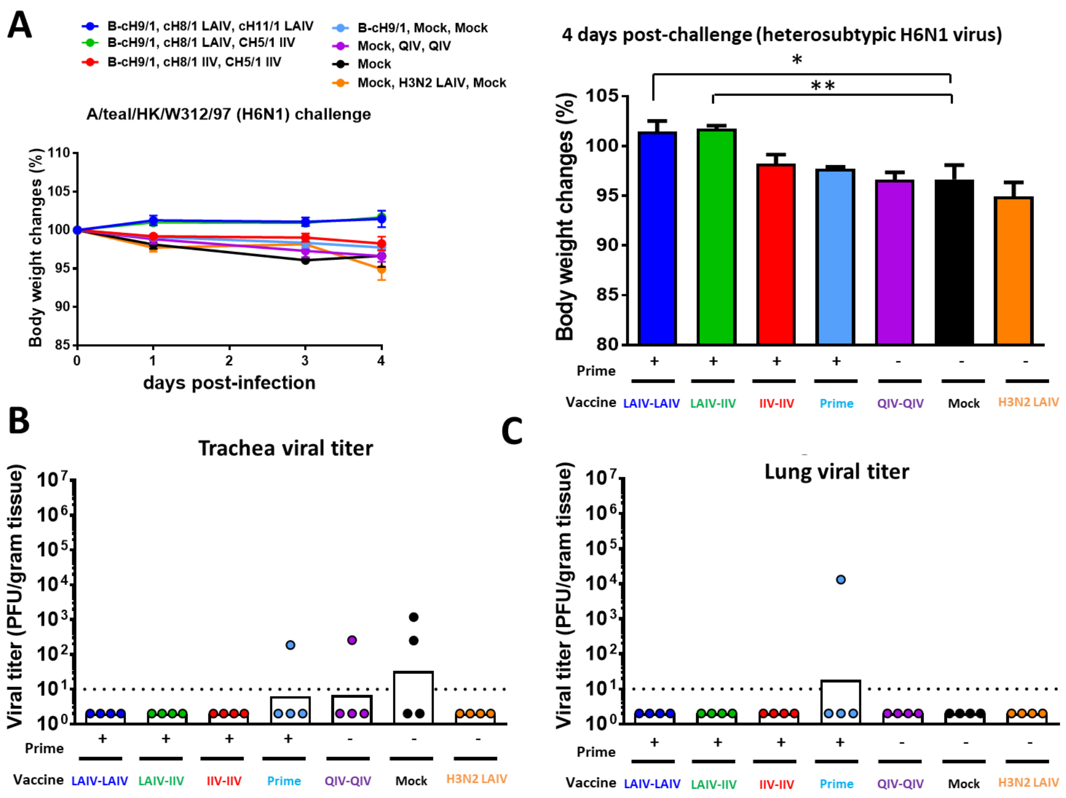

FIG.S6 Body weight changes and viral replication in the lower respiratory tract post challenge infection with H6N1 influenza virus.

(A) After challenge infection with 10^6 PFU of an H6N1 influenza virus, body weight changes for each ferret were monitored daily from day 0 to day 4. The mean ± SEM for each group is plotted. (B, C) Viral titers following challenge infection of each immunized ferret were measured by plaque assay. LAIV-LAIV vaccinated animals are shown in blue. LAIV followed by AS03-adjuvanted IIV vaccinated animals are shown in green. Two doses of AS03-adjuvanted IIV vaccinated animals are shown in red. Prime only, two doses of QIV, and single dose of H3N2 LAIV vaccinated animals are shown in light blue, purple, and orange, respectively. Mock-immunized animals are shown in black. All cHA-vaccinated ferrets were primed with influenza B-cH9/1 virus. White bars indicate the GMT with individual scatter dot plots. Each point indicates the titer for each individual ferret (n = 4/group). The black dashed line indicates the limit of detection for the assay. (B) Trachea and (C) lung viral titers were determined on day 4 post challenge infection. Data were analyzed by one-way ANOVA followed by a Tukey’s multiple comparison tests. The asterisks refer to the level of significance: *: $p<0.05$; **: $p<0.01$.

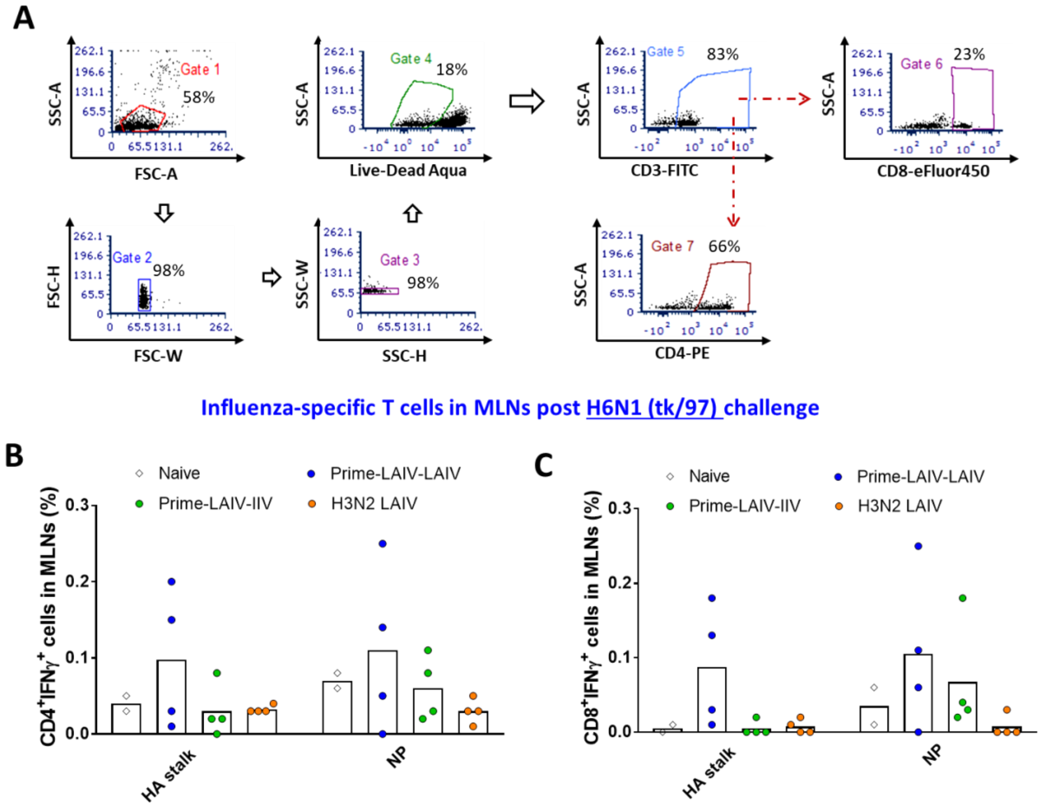

FIG. S7 Flow cytometric gating scheme and analysis of influenza-specific T cell responses in MLNs

The gating strategies for immunophenotyping of immune cells by a LSRII flow cytometer are indicated. (A) Singlets of lymphocytes (gated by SSC-A vs. FSC-A, followed by FSC-H vs. FSC-W and SSC-W vs. SSC-H), and live cells (based on Live/Dead Aqua stain) were acquired for analysis of T cell responses. $CD3^+$, $CD4^+$, and $CD8^+$ T cells were isolated by distinct fluorescent dyes. (B, C) Influenza HA stalk-specific and NP-specific responses of T cells isolated from MLNs are shown for the Prime-LAIV-LAIV (blue circles), Prime-LAIV-IIV (green circles), H3N2 LAIV (orange circles), or naïve animals (white diamonds) following challenge infection with an H6N1 influenza virus. Percentages of $CD4^+IFN\text{-}\gamma^+$ (B) and $CD8^+IFN\text{-}\gamma^+$ (C) cells were calculated and plotted on y-axis. Each point indicates the titer for each individual ferret (n = 2 or 4/group).
